# Mechanism of cancer-associated fibroblast-driven thyroid cancer dedifferentiation via the ZFP57-PKM2 axis-mediated lactate secretion and therapeutic intervention with resveratrol​

**DOI:** 10.1186/s13046-026-03675-w

**Published:** 2026-02-27

**Authors:** Xiaoyu Ji, Chengzhou Lv, Xian Wu, Hongpeng Wu, Yuang Chang, Qi Liu, Wenwu Dong, Jiapeng Huang, Dalin Zhang, Yao Diao, Dapeng Sun, Zhihong Wang, Ping Zhang, Wei Sun, Hao Zhang

**Affiliations:** 1 Department of Thyroid Surgery, The First Affiliated Hospital of China Medical University, No. 155 Nanjing North Street, Shenyang, Liaoning 110001 China; 2 Department of Urology, The First Affiliated Hospital of China Medical University, No. 155 Nanjing North Street, Shenyang, Liaoning 110001 China; 3 Department of Nuclear Medicine, The First Affiliated Hospital of China Medical University, No. 155 Nanjing North Street, Shenyang, Liaoning 110001 China; 4 Department of Hepatobiliary Surgery General Hospital of Northern Theater Command, No.83,Wenhua Road, Shenhe District, Shenyang, 110016 China

**Keywords:** Spatial transcriptomics, Papillary thyroid carcinoma, Cancer-associated fibroblasts, Dedifferentiation, Glycolysis

## Abstract

Papillary thyroid carcinoma (PTC) undergoes dedifferentiation into aggressive poorly differentiated (PDTC) or anaplastic (ATC) carcinomas in 10–15% of cases, a process potentially driven by cancer-associated fibroblasts (CAFs) within the tumor microenvironment (TME), though their spatiotemporal dynamics remain poorly understood. Resveratrol (Res), a natural compound, has shown anticancer potential by promoting redifferentiation and apoptosis while inhibiting oncogenic signaling, suggesting utility in countering PTC dedifferentiation. Using spatial transcriptomics (10× Visium) on three surgical specimens, we obtained 14,191 high-quality spots annotated via UMAP and Leiden clustering into seven cell types, including CAFs, T cells, B cells, and others. Comparative gene expression and functional enrichment analyses revealed CAFs in poorly differentiated regions exhibited heightened glycolytic activity, correlated with ZFP57 upregulation and PKM2 induction. Glycolysis was validated through immunofluorescence, Seahorse assays, glucose/lactate measurements, and ZFP57-PKM2 reporter assays. CAF-conditioned media promoted PTC proliferation, invasion, and dedifferentiation while reducing radioiodine uptake in co-culture models. In xenografts, ZFP57 overexpression increased tumor growth and impaired radioiodine retention, whereas Res suppressed ZFP57, restored differentiation, enhanced radioiodine avidity, and inhibited glycolysis. Mechanistically, CAF-secretated lactate activated TGF-β/Smad2/3 signaling, fostering dedifferentiation and malignancy. Resveratrol reversed these effects by targeting ZFP57, normalizing CAF metabolism, and restoring PTC differentiation, indicating a promising therapeutic strategy against PTC progression.

**Supplementary Information:**

The online version contains supplementary material available at 10.1186/s13046-026-03675-w.

## Introduction

Papillary thyroid carcinoma (PTC) represents the most prevalent subtype of thyroid cancer, accounting for over 80–85% of all thyroid malignancies [1]. More than 90% of PTC patients typically exhibit a favorable prognosis with a 10-year survival rate [2]. However, 10–15% of PTC cases ultimately undergo dedifferentiation into more aggressive poorly differentiated thyroid carcinoma (PDTC) or anaplastic thyroid carcinoma (ATC) [3–8]. Dedifferentiation is a biological process driving the transition of cancer cells from a well-differentiated to a poorly differentiated state [9]. Cellular plasticity, which enables cancer cells to shift between differentiated and undifferentiated states, facilitates this dedifferentiation in malignancies [10, 11]. Such phenotypic fluctuations may involve diverse genetic alterations across cancer types [12–15]. Furthermore, the hypothesis that ATC originates from PTC is supported by indirect evidence, including the coexistence of PTC components within ATC lesions and the clinical history of PTC in most ATC patients [6]. Notably, a stark survival disparity exists between ATC/PDTC and PTC. Reported 5-year survival rates for PDTC patients range from 50% to 64% [8, 16], while ATC, representing 1.7% of all thyroid cancers, exhibits a median survival of only 6 to 8 months and contributes to 33–50% of thyroid cancer-related deaths [6]. Thus, precise stratification of aggressive PTC for proactive clinical intervention is critical to prevent its progression and dedifferentiation into PDTC/ATC.

The tumor microenvironment (TME) serves as a key driver of tumor progression. Cross-talk between cancer cells and the TME sustains tumor survival, promotes invasion and metastasis, induces angiogenesis, and diminishes chemosensitivity [17–19]. Cancer-associated fibroblasts (CAFs), the predominant stromal component of the TME, originate from bone marrow-derived mesenchymal stem cells, hematopoietic stem cells, adipocytes, and endothelial cells [20, 21]. These cells secrete diverse growth factors and cytokines while degrading extracellular matrix proteins, thereby influencing tumor cell proliferation, metastasis, and chemoresistance [22–24]. Remarkably, CAFs can stably maintain pro-tumorigenic properties even in the absence of direct cancer cell exposure [25]. However, the intricate dynamics of CAF-mediated tumor dedifferentiation during thyroid cancer progression remain poorly elucidated. Advances in spatial omics technologies have facilitated the exploration of molecular differences between coexisting lesions with distinct differentiation states. Spatial transcriptomics (ST), an integrative technology combining imaging, biomarker analysis, sequencing, and bioinformatics, enables precise localization of gene expression within intact tissue sections. This approach preserves tissue architecture while revealing spatial distributions of cell types, analyzing intercellular interactions, and constructing regional gene expression profiles [26, 27]. Spatial transcriptomics has demonstrated transformative potential in characterizing the TME and deciphering cell-cell interactions [28, 29]. Consequently, this technology provides spatial visualization of immune cell distributions relative to cancer cells and links these patterns to molecular signatures driving specific immune cell infiltration.

The development of novel therapies or agents to counteract dedifferentiation-prone PTC holds significant clinical value for preventing disease progression. Resveratrol (Res), a naturally occurring polyphenol compound found in plants such as grapes and peanuts [30, 31], exhibits multifaceted biological activities. The anticancer effects and multi-target properties of this polyphenol are well-documented [32], with mechanisms including induction of redifferentiation and apoptosis, as well as inactivation of cancer-associated signaling pathways [33]. Importantly, anticancer doses of Res demonstrate minimal cytotoxic effects on normal cells [34–36]. These properties position Res as a potential therapeutic candidate for dedifferentiation in PTC. Recent studies have shown that Res induces sodium-iodide symporter (NIS) expression and enhances iodine uptake in the rat thyroid cell line FRTL-5 [37]. However, data on Res effects in dedifferentiation-prone PTC tissues and its modulation of CAFs remain limited.

In this study, we generated spatial transcriptomic sequencing data to characterize cellular compositions across PTC, PDTC, and ATC. Through integrated analyses, we investigated microenvironmental differences between PDTC/ATC and PTC. Furthermore, we employed spatial transcriptomics to delineate molecular trajectories underlying the evolution from PTC to PDTC/ATC, identifying critical genomic events driving this transition. Our work elucidates potential mechanisms of CAF-mediated PTC progression, offering insights for prognostic stratification and therapeutic strategies to reverse dedifferentiation or induce redifferentiation.

## Material and methods

### Human specimens and cell lines

This study utilized tissue specimens from two cases of coexisting PTC and PDTC, as well as one case of coexisting PTC and ATC. All specimens were obtained from patients who underwent surgical resection at the First Affiliated Hospital of China Medical University between 2014 and 2022. Formalin-fixed, paraffin-embedded (FFPE) tissues were subjected to immunohistochemical analysis, with diagnoses independently confirmed by two histopathologists. None of the patients received local or systemic therapy prior to surgery. All procedures involving human participants adhered to the ethical standards of the Institutional Review Board of the First Affiliated Hospital of China Medical University and the Declaration of Helsinki (1964) and its subsequent amendments. The human PTC cell lines (TPC1 and BCPAP) were employed in this study. Detailed sources and culture conditions are provided in Additional File 2: Supplementary Materials and Methods.

### Spatial transcriptomics workflow and data analysis

ST profiling was performed on FFPE samples using the 10x Genomics Visium platform. Tissue sections were dewaxed, stained with H&E, and processed for whole transcriptome probe hybridization, spatial barcode capture, and probe extension to construct sequencing libraries. Libraries were sequenced on the Illumina NovaSeq platform using paired-end reads following quality assessment. Raw sequencing data were demultiplexed and aligned to the GRCh38 reference genome via the Space Ranger pipeline, generating a gene expression matrix with spatial coordinates and UMI counts. Data quality control (including count thresholds and mitochondrial gene filtering) and batch effect correction using BBKNN were implemented in Scanpy. Spatial spots were annotated into seven cell types (fibroblasts, T/B cells, epithelial cells, myeloid cells, progenitor cells, and endothelial cells) based on canonical marker genes, preserving tissue architecture and spatial gene expression patterns. Full analytical protocols are described in Additional File 2: Supplementary Materials and Methods.

### Differential gene expression and functional enrichment

Fibroblast-enriched regions across three samples were isolated, and differentially expressed genes (DEGs) between poorly differentiated and well-differentiated regions were identified using the scanpy.tl.rank_genes_groups function in Scanpy. DEG lists were exported as CSV files and subjected to Gene Ontology (GO) and Kyoto Encyclopedia of Genes and Genomes (KEGG) pathway enrichment analyses with the R package clusterProfiler (version 4.10.1).

### Gene co-localization analysis

Spatial transcriptomic data in h5ad format were converted to R-compatible Seurat objects using the sceasy R package (version 0.0.7). Co-localization of target genes within fibroblast-enriched regions was quantified using the Seurat package (version 5.1.0) by tallying spots exhibiting concurrent high expression.

### Isolation and validation of CAFs

CAFs were isolated from fresh thyroid tumor tissues resected at the First Affiliated Hospital of China Medical University, with written informed consent obtained from all patients. One primary CAF line was successfully established from a single patient donor and used throughout this study. To ensure experimental consistency and minimize inter-donor variability, a master cell bank was created at early passages, and cells from the same passage (P4) were used for all conditioned medium (CM) production and subsequent experiments. Tissues were collected within 30 min post-resection, transported in DMEM (10% FBS) on ice, and minced into fragments after PBS washing. Cell suspensions were centrifuged (1000 rpm, 4 min), resuspended in fresh DMEM (10% FBS), and cultured in 100 mm dishes at 37 °C under 20% O₂ and 5% CO₂. Media were refreshed thrice weekly. CAF identity was validated by immunofluorescence staining, confirming positive expression of established CAF markers (α-SMA). CAFs at passage 4 were used for experiments.

### Conditioned medium (CM) collection

For CM collection, CAFs at 80–90% confluency were washed with PBS and incubated in serum-free DMEM for 24, 48, or 72 h. Supernatants were centrifuged (1600 rpm, 4 min), filtered through 0.22 μm membranes, and diluted 5:6 with RPMI 1640 containing 10% FBS for subsequent experiments. For all subsequent experiments involving CM treatment, PTC cells were incubated with the prepared CM for 48 h unless otherwise specified.

### Luciferase reporter assay

ZFP57 binding motifs in the PKM2 promoter were predicted via JASPAR (http://jaspar.genereg.net/). Wild-type and mutant promoter fragments (lacking EHF binding sites) were synthesized and cloned into the pGL3-Basic vector (Promega). Constructs were validated by sequencing. Cells seeded in 96-well plates were co-transfected with ZFP57 overexpression vectors and promoter constructs using Lipofectamine 3000 according to the manufacturer‘s protocol. After 6 h, the transfection mixture was replaced with fresh complete medium. Cells were then cultured for an additional 42 h (48 h post-transfection in total) before luciferase activity was measured 48 h post-transfection using the Dual-Luciferase Reporter System (Promega).

### In vitro ^131^I uptake in PTC cells

PTC cells (TPC1and BCPAP; 5 × 10^5^ cells/well in 12-well plates) were washed with cold HBSS and incubated for 30 min at 37 °C with 1 µCi carrier-free Na^131^I and 10 µM NaI in HBSS. For nonspecific uptake controls, cells were pretreated with 300 µM NaClO4 for 30 min. After washing, cells were lysed in 0.33 M NaOH, and radioactivity was quantified using a gamma counter.

### Glucose consumption and lactate production

Tissue homogenates were centrifuged to collect supernatants. Glucose and lactate levels were measured using enzymatic colorimetric kits (Nanjing Jiancheng Bioengineering Institute) by monitoring absorbance at 530 nm after incubation with specific reagents. The median lactate concentration in conditioned medium collected from unstimulated control CAFs (cultured for 48 h in serum-free DMEM) was 3.9 mM (interquartile range: 3.5–4.3 mM), as determined from three independent batches of CM preparation.

### Cellular metabolism assays

Metabolic profiling (oxygen consumption rate, OCR; extracellular acidification rate, ECAR) was performed using an XF96 Analyzer (Agilent Technologies). PTC cells (2.31 × 10^5/well) were seeded in CellTak-coated 96-well plates. OCR was measured sequentially with oligomycin (1.5 µM), FCCP (1.0 µM), rotenone (1.0 µM), and antimycin A (1.8 µM). ECAR was assessed following glucose (10 mM), oligomycin (2 µM), and 2-DG (50 mM) treatments.

### Xenograft model and ^131^I uptake

All animal procedures were approved by the Animal Care and Use Ethics Committee of China Medical University (Approval Number: [KT20250536]) and performed in accordance with the approved protocol. The maximal tumor burden permitted was 2000 mm³, and we confirm that this limit was never exceeded. TPC1 cells mixed with CAFs (1:1 ratio; 1 × 10^6 cells in 200 µL) were subcutaneously injected into BALB/c nude mice (4–5 weeks old). Starting on day 5, the treatment group (*n* = 10) received daily intraperitoneal resveratrol (100 mg/kg in 5% ethanol + 25% PEG 400), while controls received vehicle. Tumor dimensions were measured every 2 days (volume = 0.52×L×W×T). At 100–150 mm³, mice received 50 µCi Na^131I via tail vein, with controls receiving saline, NaClO4-pretreated ^131I, or no treatment. Tumors, thyroid, and muscle tissues were homogenized post-euthanasia (CO2 asphyxiation), and radioactivity (cpm/g) was quantified.

### Chromatin immunoprecipitation (ChIP)

Cells (1 × 10^6) were crosslinked with 1% formaldehyde (10 min, 37 °C), lysed in SDS buffer, and sonicated to shear DNA (200–1000 bp). After crosslink reversal (65 °C, 4 h), lysates were pre-cleared with protein A agarose/salmon sperm DNA and incubated overnight with target antibodies. Immune complexes were washed sequentially with low-salt, high-salt, LiCl, and TE buffers, eluted in SDS/NaHCO3, and purified for PCR analysis.

### Molecular docking

Protein-ligand docking was performed using AutoDock 4.2. The structure of mibefradil was retrieved from PubChem (https://pubchem.ncbi.nlm.nih.gov/), and protein crystal structures were obtained from the PDB (https://www.rcsb.org/). Ligands were extracted from complexes using PyMOL 2.3.4. Hydrogen atoms were added to receptors prior to docking.

### Statistical analysis

Data are presented as mean ± SD from at least three independent experiments. Statistical analyses were performed using GraphPad Prism 8.3 and SPSS 25.0. Comparisons between two groups used an unpaired two-tailed t-test. For multiple groups, one-way or two-way ANOVA with Tukey’s post-hoc test was applied, as detailed in figure legends. Significance levels are denoted as *p < 0.05, **p < 0.01, ***p < 0.001, with exact comparisons specified in the legends.

## Results

### Spatial transcriptomics reveals caf-mediated glycolytic regulation of tumor dedifferentiation

**Fig. 1:**
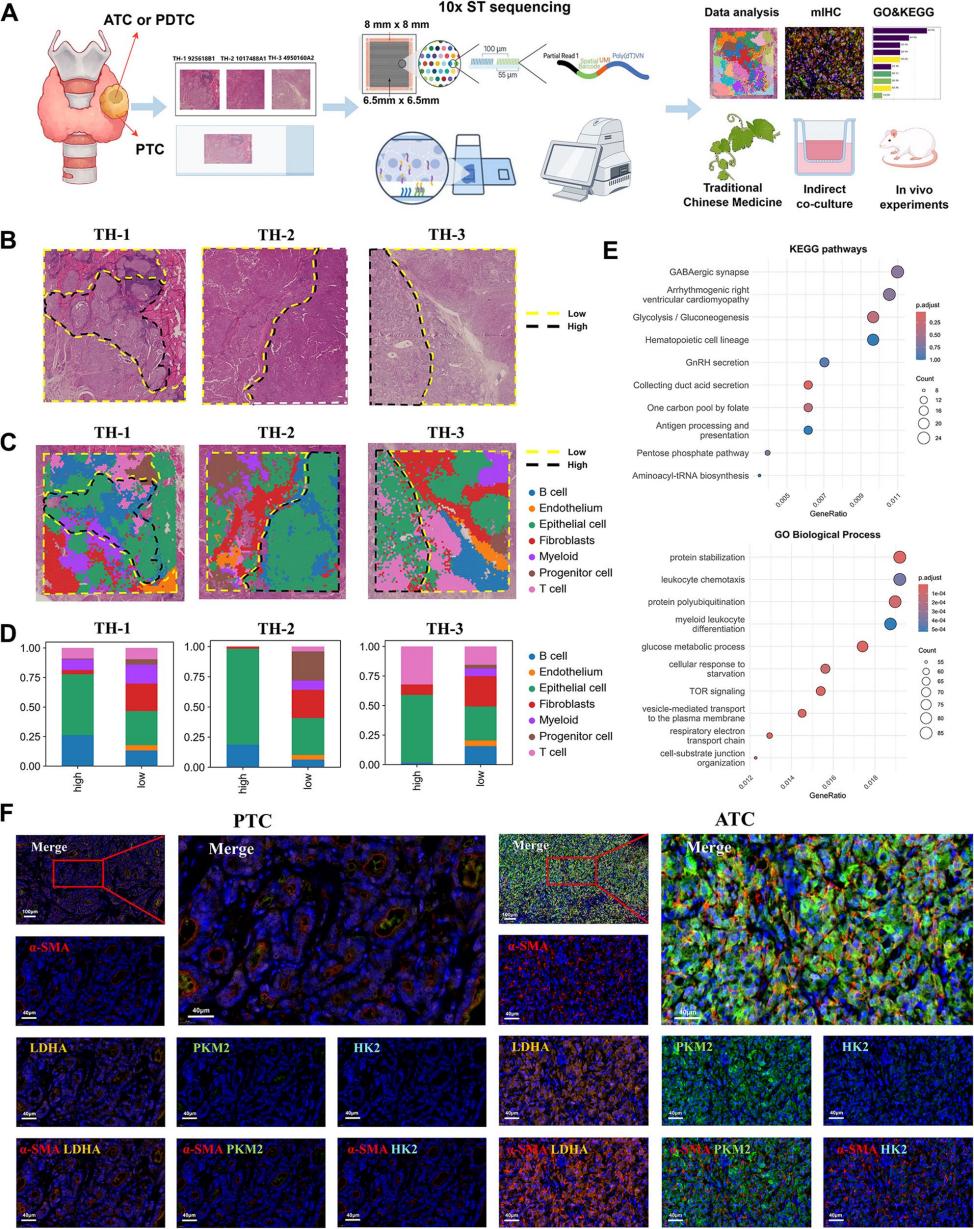
Spatial Transcriptomic Features Of Tumor Tissue And Microenvironment Analysis.** A** Sequencing analysis of three paraffin - embedded tumor samples based on the 10x Genomics Visium spatial transcriptomic technology. **B** Pathological zoning results of H&E - stained sections (Low: poorly differentiated areas, ATC/PDTC; High: well - differentiated areas, PTC), with zoning criteria referring to the WHO (2022) tissue differentiation grading guidelines. **C** Spatial distribution patterns of major cell types displayed via Space Ranger (v2.0.0) analysis, with color coding corresponding to different cell subgroups (Low: poorly differentiated areas, ATC/PDTC; High: well - differentiated areas, PTC). **D** Spatial distribution patterns of major cell types shown through bar charts, with color coding corresponding to different cell subgroups. **E** Bubble plot of GO/KEGG pathway enrichment analysis performed on the CAFs compartment extracted from spatial transcriptomics data. Bubble size represents the number of genes, and color depth indicates the -log₁₀(p-value). **F** Multiple immunofluorescence staining validation of the co - localization of CAFs (α - SMA+, red) with key glycolysis enzymes (LDHA, yellow; HK2, light blue; PKM2, green). DAPI nuclear staining (blue). The red dashed - line box indicates the locally magnified area (scale bar: main Fig. 100 μm; inset 40 μm)

ST analysis was performed on three FFPE thyroid carcinoma tissue blocks from three unique patients using the 10x Genomics Visium platform (Fig. 1A). A total of 14,191 spatially resolved transcriptomic spots were sequenced. Based on hematoxylin and eosin (H&E) staining, tissue regions were classified into PTC and ATC/PDTC zones according to distinct histopathological features (Fig. 1B). ST objects were constructed and subjected to rigorous quality control (Figure S1A-J), retaining 4567 spots (ST1), 4855 spots (ST2), and 4769 spots (ST3) for downstream analysis. Uniform Manifold Approximation and Projection (UMAP) was employed for dimensionality reduction after batch effect correction, followed by Leiden clustering, yielding 17, 18, and 18 transcriptionally distinct clusters for ST1, ST2, and ST3, respectively (Figure S2A). Using established marker genes, all spots were annotated into seven cell types: fibroblasts (COL1A2, PDGFRB, RGS5; 622, 964, and 632 spots), T cells (CD3E, CD3D, NKG7; 101, 1031, and 387 spots), B cells (MS4A1, CD79A, IGHG1; 544, 526, and 815 spots), epithelial cells (EPCAM, CLU, TG; 2406, 1872, and 2107 spots), myeloid cells (FCER1G, CSF1R, LST1; 194, 210, and 539 spots), progenitor cells (MKI67, TOP2A, STMN1; 602, 97, and 164 spots), and endothelial cells (RAMP2, VWF, PTPRB; 98, 155, and 6125 spots) (Figure S2B-D). Spatial mapping of these cell types revealed distinct distribution patterns across tissue regions (Fig. 1C). Comparative quantification of cell populations between differentiation states demonstrated that CAFs exhibited the most pronounced numerical differences between poorly differentiated (ATC/PDTC) and well-differentiated (PTC) regions (Fig. 1D, Figure S2E). CAFs from all three samples were isolated for differential gene expression analysis between dedifferentiated and differentiated zones. GO enrichment highlighted significant involvement in glucose metabolic processes, while KEGG analysis showed that the Glycolysis/Gluconeogenesis pathway was significantly enriched (Fig. 1E). Multiplex immunofluorescence (IF) staining showed that α-SMA-positive CAFs were spatially proximate to cells expressing high levels of glycolytic enzymes (GLUT1, HK2, PKM2) within the tumor stroma. This phenomenon was particularly evident in ATC/PDTC regions (Fig. 1F). Notably, this spatial proximity was markedly enhanced in ATC/PDTC zones, suggesting that CAFs within dedifferentiated regions exhibit amplified glycolytic activity. These findings collectively indicate progressive upregulation of CAF-driven glycolysis during thyroid cancer dedifferentiation.

### Upregulation of ZFP57 in CAFs correlates with glycolytic gene PKM2 and enhances glycolysis

**Fig. 2:**
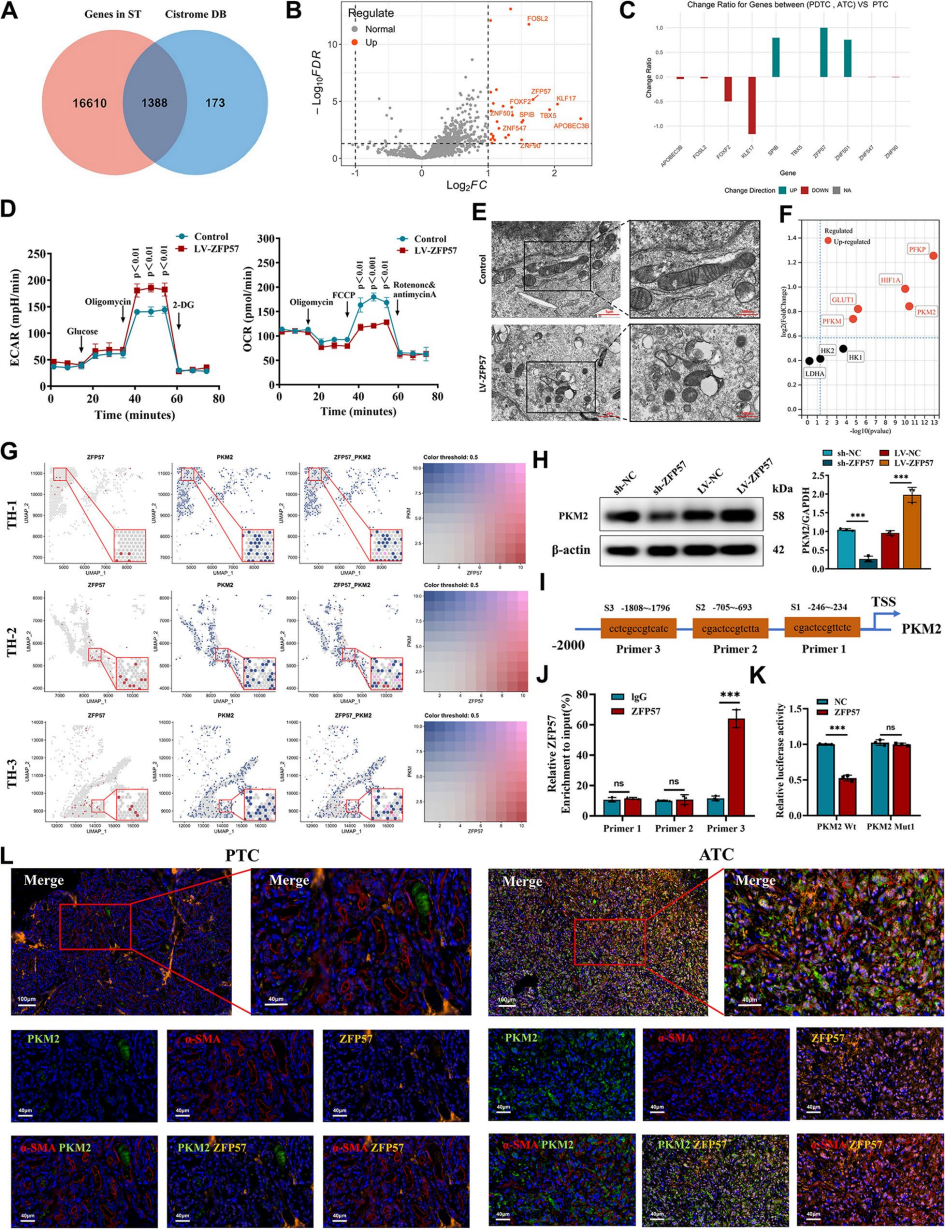
ZFP57 Regulates The Glycolytic Activity Of CAFs.** A** Identification of common transcription factors from spatial transcriptomic data (ST) and the Cistrome DB database (v4.0). **B** Volcano plot of differentially expressed transcription factors in CAFs (red: up - regulated factors, log2FC > 1; grey: no significant difference, FDR < 0.05). **C** Analysis of mRNA proportions of different types of thyroid cancer in the GEO database (GSE235468). **D** Seahorse XF96 cell energy metabolism analysis: Changes in the extracellular acidification rate (ECAR) and oxygen consumption rate (OCR) of CAFs after ZFP57 overexpression. **E** Representative TEM images of each group. The inset (black box, locally magnified area) shows morphologically altered mitochondria. Scale bars: main Fig. 1 μm; inset 500 nm. **F** Spatial transcriptomic sequencing showing the expression of glycolysis - related enzymes in CAFs in different differentiation regions. **G** Spatial co - localization analysis of ZFP57 (blue) and PKM2 (red) in the tumor microenvironment. **H** Western blot analysis measuring the expression of ZFP57. **I-K** Validation of ZFP57 binding to the PKM2 promoter: (**I**) Prediction of ZFP57 binding sites by the JASPAR database. (**J)** Validation of binding sites by ChIP-qPCR. (**K)** Validation of the effect of binding sites on promoter activity by a dual - luciferase reporter system. **L** Multiple immunofluorescence staining validation of the localization of CAFs (α- SMA+, red), the key glycolytic enzyme PKM2 (green), and the transcription factor ZFP57 (yellow). DAPI nuclear staining (blue). The red dashed - line box indicates the locally magnified area (scale bar: main Fig. 100 μm; inset 40 μm)., For in vitro experiments or cell samples, *n* ≥ 3 for each group. Data are mean ± SD. *p < 0.05, **p < 0.01, and ***p < 0.001. Statistical significance was determined by one-way ANOVA or two-sided Student’s t-test as appropriate

Previous studies highlight transcription factors (TFs) as central regulators orchestrating downstream gene networks in cancer, offering therapeutic potential through multi-pathway modulation [38, 39]. However, their context-dependent roles in thyroid cancer remain underexplored. To investigate CAF-driven mechanisms in PTC, we first utilized spatial transcriptomics (ST) data to identify all differentially expressed genes (DEGs) in CAFs between low-differentiation (ATC/PDTC) and high-differentiation (PTC) regions. This complete set of DEGs was then intersected with the CistromeDB transcription factor database, which identified 1,388 genes that were both differentially expressed and annotated as transcription factors (Fig. 2A). Volcano plot analysis of differentially expressed genes in CAFs identified and ranked the top 10 differentially expressed TFs: APOBEC3B, TBX5, KLF17, FOSL2, ZNF501, SPIB, ZNF547, ZNF90, FOXF2, and ZFP57 (Fig. 2B). Validation using the bulk RNA-seq dataset GSE235468 confirmed upregulation of ZFP57, ZNF501, and SPIB in PDTC/ATC tissues (Fig. 2C). Notably, several CAF-upregulated TFs from spatial transcriptomics (e.g., APOBEC3B, TBX5) were not elevated in bulk data, likely due to cell-type specificity: spatial analysis captures CAF-enriched signals from dedifferentiated niches, whereas bulk tissue dilutes these stromal-specific changes. This underscores the necessity of resolving stromal–epithelial compartments to identify genuine CAF-driven regulators.Primary CAFs isolated from fresh tumor samples (Figure S3A) were used to screen for effective knockdown constructs. Three independent shRNAs targeting each of the transcription factors ZNF501, SPIB, and ZFP57 were individually evaluated. Their knockdown efficiencies were first assessed by q-PCR (Figure S3B), followed by confirmation at the protein level via Western blotting (Figure S3C). Based on this combined analysis, the most potent shRNA for each gene (sh-ZNF501-1, sh-SPIB-2, and sh-ZFP57-2) was selected for all subsequent experiments. Extracellular lactate and glucose analyses revealed that silencing ZFP57, ZNF501, or SPIB significantly reduced glucose uptake and lactate production in CAFs, with ZFP57 knockdown showing the strongest effect (Figure S3D). Conversely, ZFP57 overexpression (Figure S3E-F) markedly increased glucose consumption and lactate output (Figure S3G). Seahorse XF metabolic assays revealed that ZFP57 overexpression significantly suppressed the maximal oxygen consumption rate (OCR, induced by FCCP) while elevating the maximal extracellular acidification rate (ECAR, induced by oligomycin) (Fig. 2D), indicating constrained mitochondrial respiratory capacity and enhanced glycolytic potential. Correspondingly, transmission electron microscopy further demonstrated that ZFP57 overexpression induced alterations in mitochondrial structure (Fig. 2E). In contrast, ZFP57 knockdown produced the opposing effects on these maximal metabolic parameters (Figure S3H) but did not induce significant alterations in mitochondrial structure (Figure S3I). Glycolysis involves key enzymes such as HIF-1α, HK1, HK2, LDHA, PFKM, PFKP, PKM2, and GLUT1. Using ST data, we compared the expression levels of these enzymes in CAFs between highly differentiated and poorly differentiated regions (Fig. 2F), followed by screening based on their spatial co-localization with ZFP57 within the same ST dataset (Fig. 2G, S4A–H). We found that PFKP, HIF-1α, and PKM2 were significantly upregulated in CAFs within dedifferentiated regions and exhibited strong spatial correlation with ZFP57. However, qPCR analysis revealed that only PKM2 expression was significantly altered following ZFP57 knockdown or overexpression, whereas changes in PFKP and HIF-1α were not statistically significant (Figure S5A). This PKM2-specific regulation was further confirmed at the protein level by Western blot analysis after ZFP57 manipulation (Fig. 2H). These results indicate that PKM2 is a primary downstream transcriptional target of ZFP57 in CAFs.JASPAR database analysis predicted three potential ZFP57 binding sites within the PKM2 promoter (Fig. 2I). Chromatin immunoprecipitation (ChIP) and luciferase reporter assays confirmed ZFP57 binding to the PKM2 promoter, driving its transcriptional activation (Fig. 2J-K). Multiplex immunofluorescence staining revealed a strong spatial association between ZFP57-expressing CAFs and elevated PKM2 levels in ATC compared to PTC tissues (Fig. 2L). This consistent spatial proximity highlights the activation of the ZFP57-PKM2 axis within the tumor stromal compartment as a key feature of dedifferentiated tumors. These findings establish ZFP57 as a critical regulator of CAF-mediated glycolysis via PKM2, promoting metabolic reprogramming during thyroid cancer dedifferentiation.

### CAF-conditioned medium promotes PTC cell proliferation and dedifferentiation

**Fig. 3:**
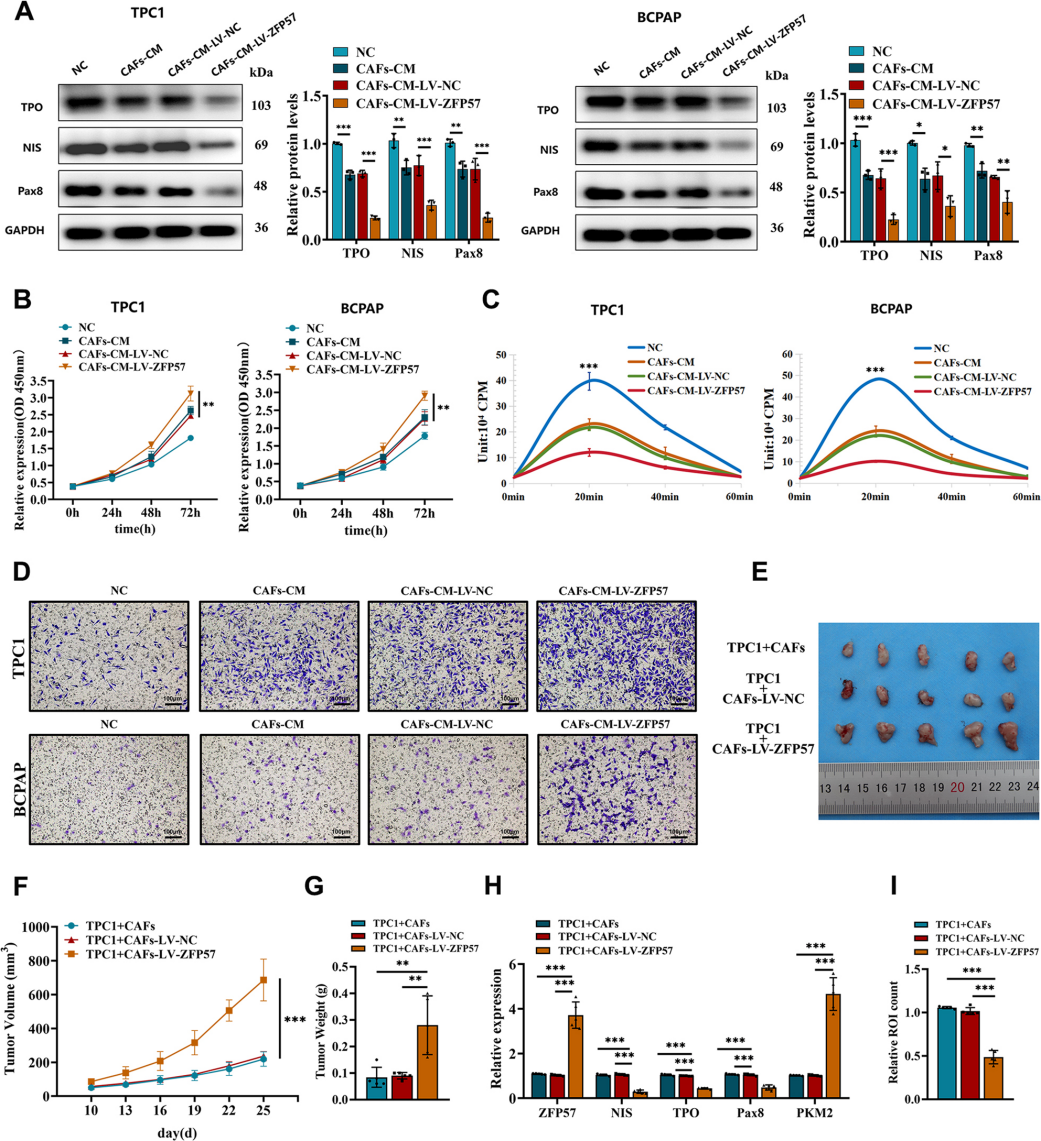
ZFP57 regulates the dedifferentiation process of thyroid cancer cells through metabolic reprogramming. **A** Western blot analysis measuring the expression of thyroid differentiation proteins (NIS, TPO, and PAX8) in TPC1 and BCPAP cells treated with conditioned media from different kinds of CAFs. **B** CCK-8 assay measuring the proliferation of TPC1 and BCPAP cells treated with conditioned media from different kinds of CAFs. **C** Radioactive iodine uptake in TPC1 and BCPAP cells treated with conditioned media from different kinds of CAFs. **D** Transwell assay measuring the invasive capacity of TPC1 and BCPAP cells treated with conditioned media from different kinds of CAFs (scale bar: 100 μm). **E** Schematic diagram of subcutaneous xenograft tumor model construction (TPC1 cells mixed with CAFs at a ratio of 1:1 for inoculation, total cell number 5 × 10⁶ per mouse, BALB/c nude mice, 6 - weeks - old). **F-I** Transplant tumor phenotype analysis: (**F**) Dynamic monitoring of tumor volume. (**G) **Ex - vivo tumor weight statistical analysis. (**H)** qRT - PCR detection of ZFP57/PKM2 signaling axis and thyroid differentiation markers in xenografts. (**I)** Ex - vivo radioactivity of ^131^I in xenograft tumor tissues. For in vitro experiments or cell samples, *n* ≥ 3 for each group. Data are mean ± SD. *p < 0.05, **p < 0.01, and ***p < 0.001. Statistical significance was determined by one-way ANOVA or two-sided Student’s t-test as appropriate

To specifically investigate the paracrine effects of CAF-secreted factors on PTC cells, independently of direct cell-cell contact, we utilized a conditioned medium (CM)-based approach. PTC cells (TPC1 and BCPAP) were treated with varying dilutions of CAF-conditioned medium (CAFs-CM). The 5/6 dilution of CAFs-CM exerted the most significant proliferative effect (Figure S5B). Initial screening of thyroid-specific genes identified PAX8, NIS, and TPO as the most markedly downregulated markers in PTC cells cultured with CAFs-CM (Figure S5C). Western blot analysis revealed that, compared to the NC group (PTC cells alone), conditioned medium from control CAFs (CAFs-CM) induced a modest decrease in NIS, TPO, and PAX8 protein levels. Importantly, this suppressive effect was significantly enhanced in cells treated with conditioned medium from ZFP57-overexpressing CAFs (CAFs-CM-LV-ZFP57), exhibiting a statistically significant reduction relative to the CAFs-CM-LV-NC group.(Fig. 3A). CCK-8 assays demonstrated that conditioned media from ZFP57-overexpressing CAFs significantly enhanced tumor cell proliferation (Fig. 3B). Conversely, radioactive iodine uptake capacity in PTC cells decreased upon exposure to conditioned media from ZFP57-overexpressing CAFs (Fig. 3C, S5D). Transwell invasion assays confirmed that conditioned media from ZFP57-overexpressing CAFs robustly promoted PTC cell migration (Fig. 3D, S5E). In a subcutaneous xenograft model, co-injection of TPC1 cells with ZFP57-overexpressing CAFs (which constitutes a direct co-culture in vivo) resulted in significantly larger tumor volumes and weights compared to control CAFs. (Fig. 3E-G). qRT-PCR analysis of xenograft tissues revealed that tumors exposed to ZFP57-overexpressing CAFs exhibited elevated PKM2 mRNA levels and reduced mRNA levels of NIS, TPO, and PAX8. (Fig. 3H). Furthermore, radioactive iodine uptake in the ZFP57-overexpressing group was markedly diminished (Fig. 3I), consistent with enhanced dedifferentiation and loss of iodine-avidity in vivo. These data collectively demonstrate that ZFP57-driven glycolytic reprogramming in CAFs promotes PTC proliferation, migration, and dedifferentiation while impairing iodine uptake capacity.

### CAFs Promote Tumor Cell Dedifferentiation via Lactate Secretion

**Fig. 4:**
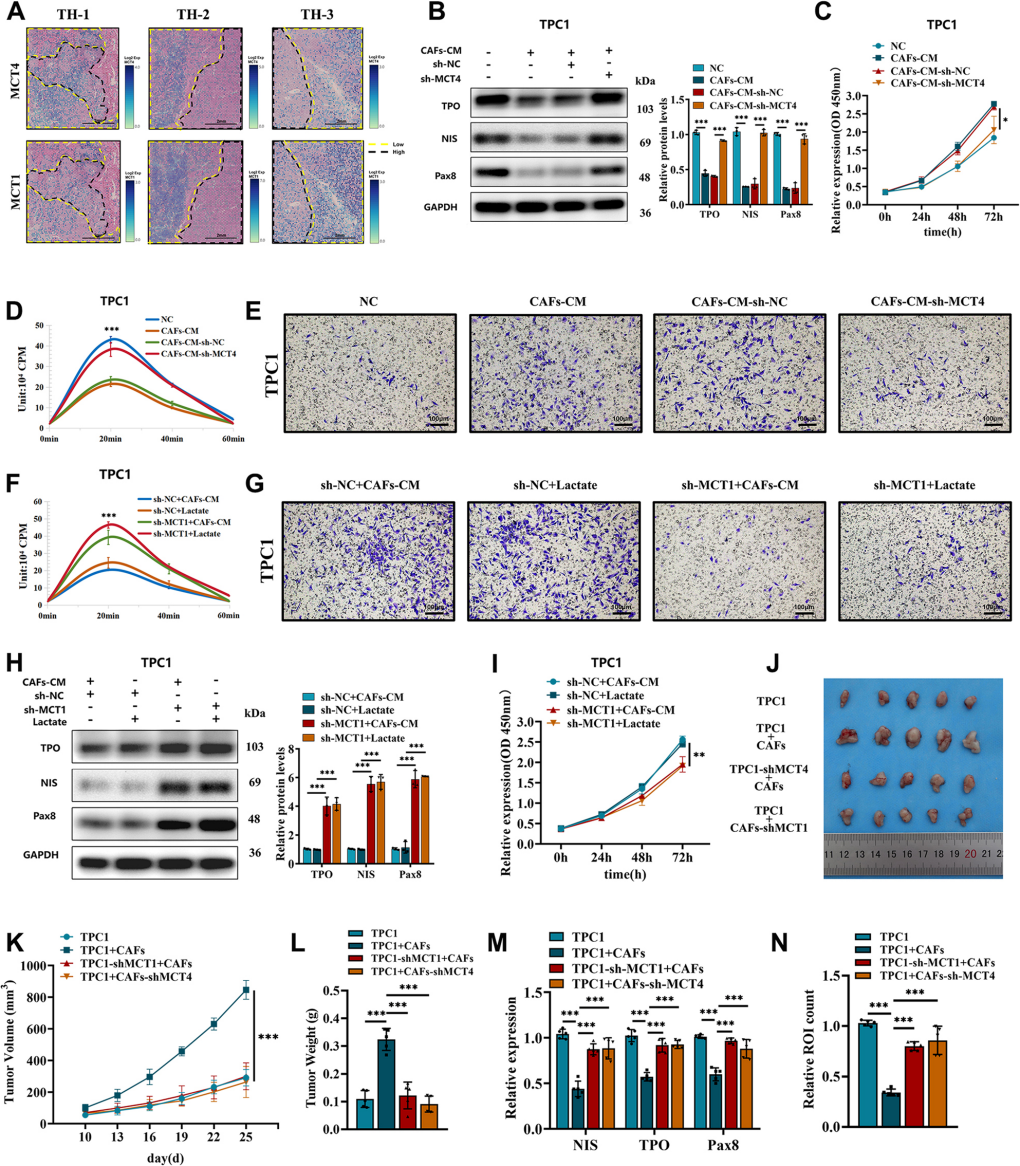
Lactate transport mediated by MCT1/MCT4 drives thyroid cancer dedifferentiation.** A** Spatial transcriptomic analysis reveals the differential expression of monocarboxylate transporters MCT1/MCT4 (blue) in low/high differentiated regions (Low: poorly differentiated areas, ATC/PDTC; High: well - differentiated areas, PTC). **B** Western blot analysis measuring the expression of thyroid differentiation proteins (NIS, TPO, and PAX8) in TPC1 cells treated with conditioned media from different kinds of CAFs. **C** CCK-8 assay measuring the proliferation of TPC1 cells treated with conditioned media from different kinds of CAFs. **D** Radioactive iodine uptake in TPC1 cells treated with conditioned media from different kinds of CAFs. **E** Transwell assay measuring the invasive capacity of TPC1 cells treated with conditioned media from different kinds of CAFs (scale bar: 100 μm). **F-I** Lactate regulates the malignant phenotype of TPC1 cells via MCT1: (**F**) Radioactive iodine uptake in TPC1 cells under different conditions is affected by treatment with CAFs-CM or lactate. **G** Transwell assay measuring the invasive capacity of TPC1 cells under different conditions is affected by treatment with CAFs-CM or lactate (scale bar: 100 μm). **H** Western blot analysis measuring the expression of thyroid differentiation proteins (NIS, TPO, and PAX8) in TPC1 cells under different conditions is affected by treatment with CAFs-CM or lactate. **I** CCK-8 assay measuring the proliferation of TPC1 cells under different conditions is affected by treatment with CAFs-CM or lactate. **J** Schematic diagram of subcutaneous xenograft tumor model construction (TPC1 cells mixed with CAFs at a ratio of 1:1 for inoculation, total cell number 5 × 10⁶ per mouse, BALB/c nude mice, 6 - weeks - old). **K-N** Transplant tumor phenotype analysis: (**K**) Dynamic monitoring of tumor volume. (**L)** Ex - vivo tumor weight analysis. (**M)** qRT - PCR analysis of thyroid differentiation markers in xenografts. (**N)** Ex - vivo radioactivity of ¹³¹I in xenograft tumor tissues. For in vitro experiments or cell samples, *n* ≥ 3 for each group. Data are mean ± SD. *p < 0.05, **p < 0.01, and ***p < 0.001. Statistical significance was determined by one-way ANOVA or two-sided Student’s t-test as appropriate

Studies suggest that CAFs undergo glycolytic reprogramming and secrete lactate, a process mediated by monocarboxylate transporters MCT1 and MCT4 (encoded by SLC16A1 and SLC16A3, respectively) [40, 41]. Spatial transcriptomics revealed significant upregulation of MCT1 and MCT4 in poorly differentiated regions compared to well-differentiated areas (Fig. 4A). Extracellular lactate analysis demonstrated that MCT4 knockdown in CAFs markedly reduced lactate secretion (Figure S6A). Compared to cells treated with control CAF-CM, PTC cells cultured with conditioned medium from MCT4-silenced CAFs (shMCT4-CM) exhibited significantly increased expression of differentiation-related proteins (PAX8, NIS, TPO) (Fig. 4B, S6B) and attenuated proliferation (Fig. 4C, S6C). Furthermore, shMCT4-CM prevented the suppression of radioactive iodine uptake (Fig. 4D, S6D) and inhibited the enhanced invasion (Fig. 4E, S6E) that were induced by control CAF-CM in PTC cells. Following a concentration-screen to determine the effective dose, exogenous lactate supplementation at 7.5 mM mimicked CAF-CM effects and significantly enhanced PTC cell proliferation (Figure S6F). However, stable MCT1 knockdown in PTC cells abolished lactate- or CAF-CM-mediated reductions in iodine uptake (Fig. 4F, S6G) and invasion (Fig. 4G, S6H). Similarly, MCT1 silencing restored differentiation markers (Fig. 4H, S6I) and suppressed proliferation (Fig. 4I, S6J), confirming that lactate uptake via MCT1 drives dedifferentiation. In vivo, xenograft tumors with MCT1- or MCT4-silenced cells (shMCT1, shMCT4) exhibited smaller volumes and weights compared to TPC1 and TPC1 + CAFs groups (Fig. 4J-L). qRT-PCR analysis revealed elevated mRNA expression of NIS, TPO, and PAX8 in shMCT4 tumors. (Fig. 4M). This upregulation was further confirmed at the protein level by Western blot analysis (Figure S6K). Notably, in the co-culture model, MCT4-knockdown in CAFs and MCT1-knockdown in tumor cells collectively led to a significant reduction in iodine avidity (Fig. 4N), consistent with restored differentiation and suppressed glycolysis. These findings establish that CAF-derived lactate, transported via MCT1/MCT4, fuels PTC dedifferentiation by impairing thyroid-specific function and enhancing aggressiveness. Targeting lactate transport may offer therapeutic strategies to reverse tumor progression.

### Lactate Stimulates Smad2/3 Phosphorylation to Drive Dedifferentiation, Proliferation, and Invasion

**Fig. 5:**
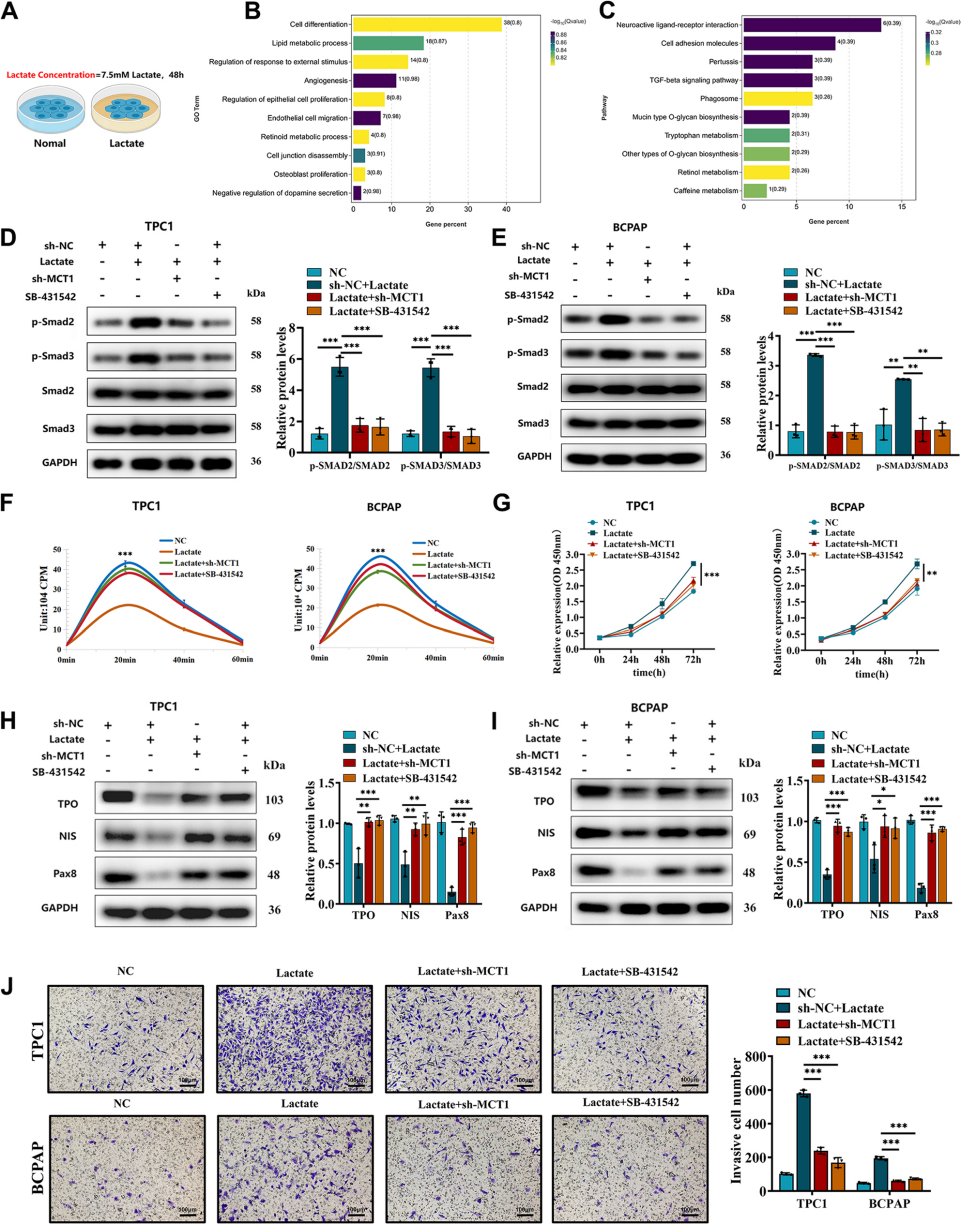
Lactate-TGFβ/smad signaling axis mediates the dedifferentiation of thyroid cancer cells.** A** Experimental setup for exogenous lactate treatment (7.5 mM lactate, simulating the tumor microenvironment for 48 h, *n* = 3 biological replicates). **B** GO pathway enrichment analysis of differentially expressed genes from RNA - seq. **C** KEGG pathway enrichment analysis of differentially expressed genes from RNA - seq. **D** Western blot analysis measuring the expression of p-Smad2/3 and Smad2/3 in TPC1 cells under different conditions is affected by treatment with lactate. **E** Western blot analysis measuring the expression of p-Smad2/3 and Smad2/3 in BCPAP cells under different conditions is affected by treatment with lactate. **F** Radioactive iodine uptake in TPC1 and BCPAP cells under different conditions is affected by treatment with lactate. **G** CCK-8 assay measuring the proliferation of TPC1 and BCPAP cells under different conditions is affected by treatment with lactate. **H** Western blot analysis measuring the expression of NIS, TPO, and PAX8 in TPC1 cells under different conditions is affected by treatment with lactate. **I** Western blot analysis measuring the expression of NIS, TPO, and PAX8 in BCPAP cells under different conditions is affected by treatment with lactate. **J** Transwell assay measuring the invasive capacity of TPC1 and BCPAP cells under different conditions is affected by treatment with lactate (scale bar: 100 μm). For in vitro experiments or cell samples, *n* ≥ 3 for each group. Data are mean ± SD. *p < 0.05, **p < 0.01, and ***p < 0.001. Statistical significance was determined by one-way ANOVA or two-sided Student’s t-test as appropriate

To investigate the mechanism of lactate-induced tumor cell dedifferentiation, RNA sequencing was performed on TPC1 cells treated with lactate for 48 h (Fig. 5A). A total of 113 differentially expressed genes (DEGs) were identified (54 upregulated and 59 downregulated; *P* < 0.05, |log2FC| >2). Subsequent GO analysis revealed significant enrichment of differentially expressed genes (DEGs) in cell differentiation-related pathways (Fig. 5B). KEGG analysis highlighted TGF-β signaling as the top enriched pathway, with Smad2/3 identified as the key nuclear effectors (Fig. 5C). Independent analysis of the upregulated and downregulated gene sets further reinforced these findings, with both subsets showing convergent enrichment in these key pathways (Figure S7A-D).To validate these pathways in a physiological tissue context, we performed Gene Set Enrichment Analysis (GSEA) using the epithelial compartment data from our spatial transcriptomics. The results confirmed significant enrichment of both the cell differentiation and TGF-β signaling gene signatures in situ (FDR < 0.05; Figure S7E), thereby providing orthogonal support for the mechanisms identified through in vitro lactate stimulation. To validate this, PTC cells were treated with lactate in the presence or absence of the TGF-β pathway inhibitor SB-431,542. Lactate treatment significantly increased Smad2/3 phosphorylation compared to controls. This effect was reversed by MCT1 knockdown or SB-431,542 co-treatment (Fig. 5D-E), confirming lactate-dependent activation of the Smad2/3 pathway. Lactate exposure significantly suppressed radioactive iodine uptake in PTC cells, an effect that was rescued by either MCT1 knockdown or TGF-β pathway inhibition (Fig. 5F, S7F). In functional assays, lactate robustly enhanced PTC cell proliferation, whereas MCT1 knockdown or TGF-β inhibition attenuated this pro‑proliferative effect (Fig. 5G). At the molecular level, lactate downregulated the mRNA and protein expression of key thyroid differentiation markers (NIS, TPO, PAX8). Conversely, MCT1 silencing or treatment with the TGF‑β receptor inhibitor SB‑431,542 restored their expression levels (Fig. 5H‑I). Finally, lactate potently promoted PTC cell invasion, which was again suppressed by MCT1 knockdown or TGF‑β pathway blockade (Fig. 5J).These findings establish that lactate promotes dedifferentiation, proliferation, and invasion in PTC via TGF-β/Smad2/3 signaling. Targeting lactate transport (MCT1) or TGF-β signaling may restore differentiation and suppress malignant progression.

### Resveratrol suppresses caf-mediated glycolysis and reverses tumor cell dedifferentiation​​

**Fig. 6:**
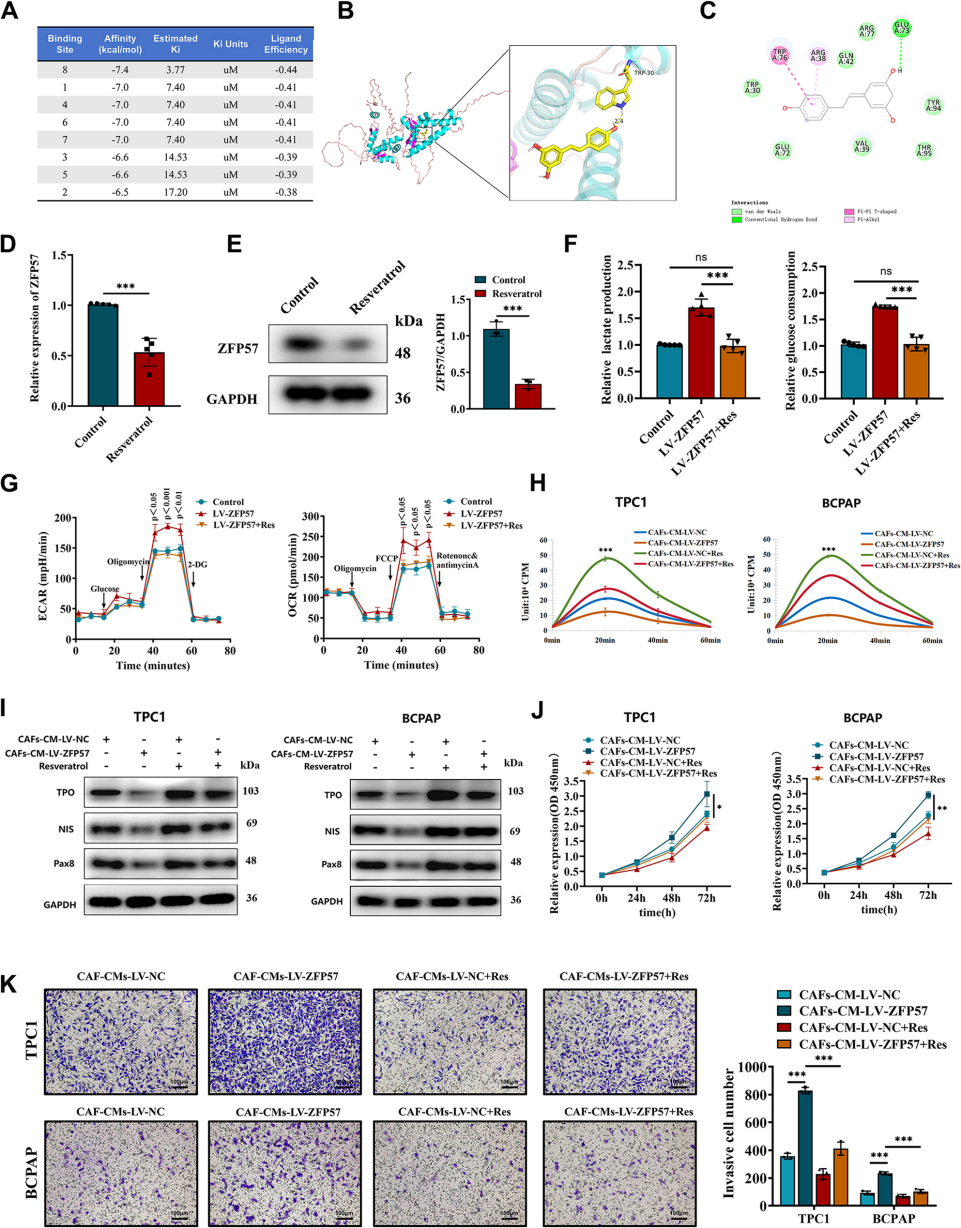
Resveratrol Targets ZFP57 to reverse metabolic abnormalities and restore thyroid cancer dedifferentiation.** A** Molecular docking of resveratrol with ZFP57 protein via AutoDock Vina reveals their binding conformation. **B**,** C** Key binding - site analysis of molecular docking: (**B**) 3D structure showing hydrogen - bonding in teractions and (**C**) 2D interaction diagram displaying π-π stacking and hydrophobic effects. **D **qRT - PCR quantifies glycolysis - related enzyme expression in CAFs after resveratrol treatment. **E** Western blot analysis measuring the expression of ZFP57 in CAFs cells treated with resveratrol. **F** Glucose uptake and lactate production assays show glycolysis in CAF cells under different conditions is affected by treatment with resveratrol. **G** Seahorse metabolic analysis shows changes in OCR and ECAR in CAF cells under different conditions is affected by treatment with resveratrol. **H** Radioactive iodine uptake in TPC1 and BCPAP cells treated with conditioned medium from CAFs that were pretreated with resveratrol under different conditions. **I** Western blot analysis measuring the expression of NIS, TPO, and PAX8 in TPC1 and BCPAP cells treated with conditioned medium from CAFs that were pretreated with resveratrol under different conditions. **J** CCK-8 assay measuring the proliferation of TPC1 and BCPAP cells treated with conditioned medium from CAFs that were pretreated with resveratrol under different conditions. **K** Transwell assay measuring the invasive capacity of TPC1 and BCPAP cells treated with conditioned medium from CAFs that were pretreated with resveratrol under different conditions (scale bar: 100 μm). For in vitro experiments or cell samples, *n* ≥ 3 for each group. Data are mean ± SD. *p < 0.05, **p < 0.01, and ***p < 0.001. Statistical significance was determined by one-way ANOVA or two-sided Student’s t-test as appropriate

**Fig. 7:**
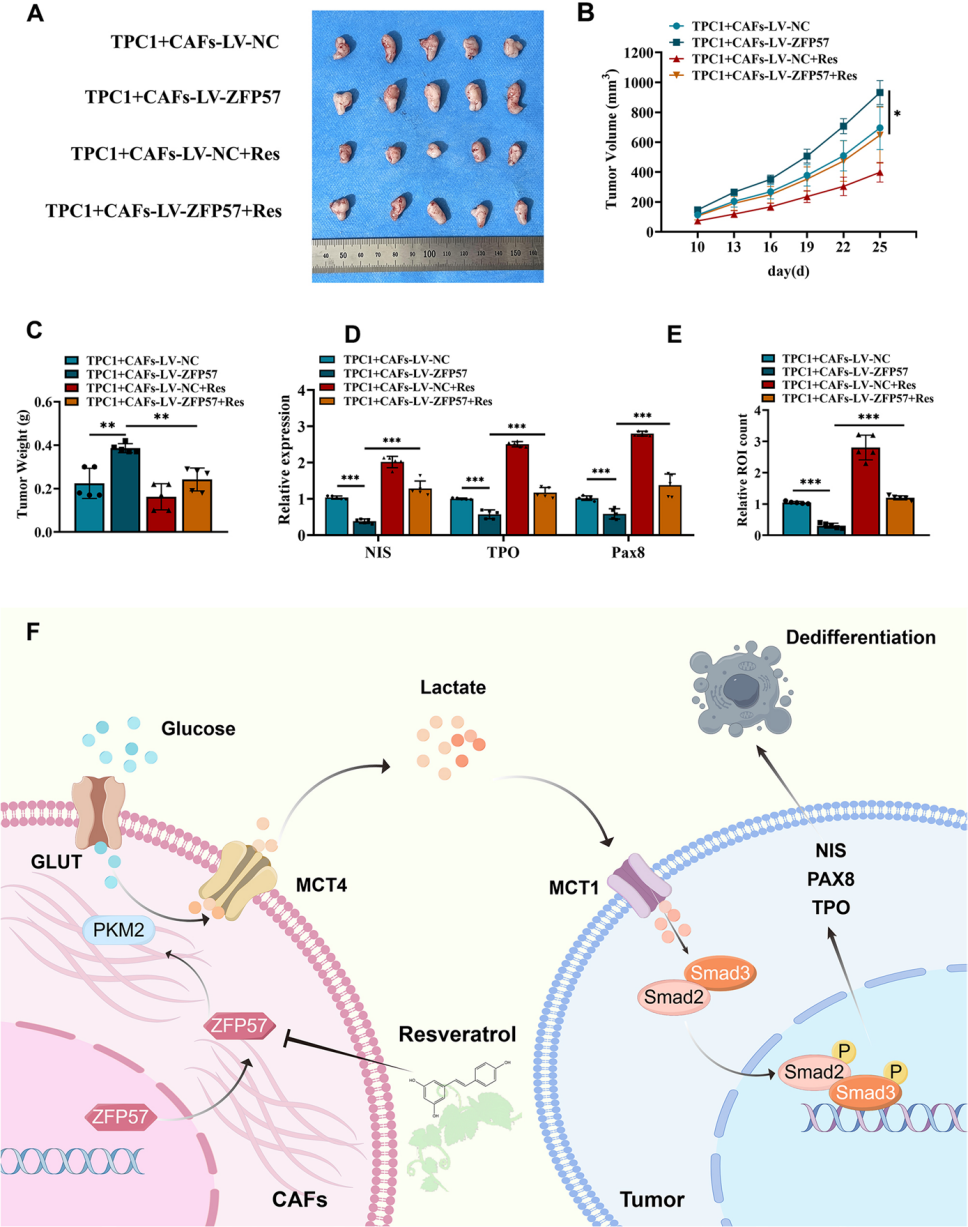
In vivo experiments targeting the ZFP57 metabolic axis to restore thyroid differentiation.** A** Schematic of subcutaneous xenograft model (TPC1 cells mixed 1:1 with CAFs, 5 × 10⁶ cells/mouse, BALB/c nude mice, aged 6 weeks). **B - E** Transplant tumor analysis: (**B**) Tumor volume monitoring. (**C)** Ex - vivo tumor weight. (**D)** qRT - PCR of thyroid differentiation markers in xenografts. (**E)** Ex - vivo ¹³¹I radioactivity in tumor tissues. **F** Mechanistic model: The ZFP57 - PKM2 glycolysis axis in CAFs drives thyroid cancer dedifferentiation via a lactate - MCT1/TGF - β/Smad signaling loop. For in vitro experiments or cell samples, *n* ≥ 3 for each group. Data are mean ± SD. *p < 0.05, **p < 0.01, and ***p < 0.001. Statistical significance was determined by one-way ANOVA or two-sided Student’s t-test as appropriate

Previous studies suggest that Res promotes redifferentiation of ATC cells, potentially reversing PTC dedifferentiation [42, 43]. To explore this, molecular docking analysis revealed potential binding sites between Res and the ZFP57 protein (Fig. 6A-C). Dose-response experiments identified 25 µM as the optimal Res concentration for suppressing lactate production in CAFs (Figure S7A-B). Res treatment significantly downregulated ZFP57 expression in CAFs (Fig. 6D-E) and attenuated glucose uptake and lactate output in ZFP57-overexpressing CAFs (Fig. 6F). Seahorse XF metabolic assays revealed that, compared to untreated ZFP57-overexpressing CAFs, Res treatment significantly enhanced the FCCP-induced maximal OCR and suppressed the oligomycin-induced maximal ECAR (Fig. 6G).This functional reversal of the metabolic phenotype was further supported by transmission electron microscopy, which showed a restoration of mitochondrial morphology (Figure S8C). Together, these data indicate that Res treatment reversed both the constrained mitochondrial respiratory capacity and the enhanced glycolytic potential induced by ZFP57 overexpression. In PTC cells treated with conditioned medium from ZFP57-overexpressing CAFs, Res treatment enhanced radioactive iodine uptake in PTC cells (Fig. 6H, S8D) and upregulated differentiation markers (NIS, TPO, PAX8) (Fig. 6I, S8E), indicating a rescue of thyroid-specific function from ZFP57-overexpressing CAF-induced suppression. In addition to restoring differentiation, Res treatment also counteracted the pro‑proliferative and pro‑invasive effects of LV-ZFP57 CAF-CM, as confirmed by CCK8 and Transwell assays (Fig. 6J-K). In vivo, Res-treated xenograft mice exhibited significantly reduced tumor volumes (Fig. 7A-B) and weights (Fig. 7C), alongside elevated NIS, TPO, and PAX8 expression (Fig. 7D) and diminished iodine avidity (Fig. 7E).These results delineate a mechanism whereby ZFP57 drives CAF glycolysis via PKM2 activation, promoting lactate secretion and TGF-β/Smad2/3-dependent tumor dedifferentiation. Resveratrol counteracts this by targeting the ZFP57-PKM2 axis, restoring CAF metabolic quiescence and enhancing tumor cell differentiation (Fig. 7F).

## Discussion

In recent years, with deepening investigations into DTC, the regulatory networks governing dedifferentiation mechanisms at pre-transcriptional, transcriptional, and translational levels have been progressively elucidated. Notably, clinical evidence indicates that a substantial proportion of PDTC and ATC originate from malignant progression of PTC [8]. This study innovatively integrates spatial transcriptomics with functional validation experiments, elucidating for the first time the cascade mechanism through which CAFs drive tumor glycolytic metabolism via the ZFP57-PKM2 regulatory axis. The secreted lactate from CAFs was further demonstrated to induce phenotypic dedifferentiation of thyroid cancer cells by activating the TGF-β/Smad2/3 signaling pathway. Furthermore, our study demonstrated that the natural compound resveratrol could effectively reverse tumor metabolic reprogramming and alleviate differentiation inhibition by targeting this signaling axis. These findings not only systematically reveal the crucial role of stromal microenvironment-tumor cell interactions in thyroid cancer dedifferentiation, but also provide both theoretical foundation and potential therapeutic targets for optimizing clinical differentiation therapy strategies.

This study innovatively employs ST to precisely map interaction zones between CAFs and tumor cells, systematically elucidating the spatiotemporal dynamics of the TME during PTC dedifferentiation. Unlike traditional sequencing methods constrained by bulk cell averaging or single-cell approaches lacking spatial resolution, ST successfully reconstructed the spatial trajectory of PTC dedifferentiation, revealing significant CAFs enrichment in poorly differentiated regions. Concurrently observed increases in myeloid and endothelial cells, though less prominent than that of CAFs, suggest a coordinated remodeling of the tumor microenvironment that may contribute to immune modulation and angiogenesis during dedifferentiation. This finding aligns with Wen et al. [44], who reported an inverse correlation between CAFs abundance and thyroid cancer differentiation status, while further extending the mechanistic link between CAFs-mediated metabolic reprogramming and tumor progression. Notably, glycolytic activity in CAFs intensified progressively with dedifferentiation, showing striking colocalization with the metabolic enzyme PKM2 in poorly differentiated niches. Metabolically, we demonstrate that lactate secreted by CAFs through the “reverse Warburg” effect not only fuel tumor cells but directly suppress organ-specific functionality—radioactive iodine uptake. This thyroid-specific mechanism diverges markedly from reported TME crosstalk in other malignancies: breast cancer CAFs induce stemness via the lactate-HIF-1α axis [45], while pancreatic cancer exhibits stroma-tumor metabolic symbiosis [46, 47]. Intriguingly, CAFs metabolic reprogramming displays both conservation and organotypic heterogeneity across cancers: ovarian malignancies rely on fatty acid oxidation-driven metastatic niches [48], breast cancer exploits glutaminolysis for invasion [49], whereas glycolytic dominance in thyroid cancer functionally contrasts with the PPP-mediated antioxidant defense in renal cell carcinoma [50].

Our study reveals the dual biological functions of ZFP57 in tumor microenvironment through demonstrating its upregulation in CAFs and positive correlation with PKM2. Previous investigations have demonstrated tissue-specific roles of ZFP57 in malignant tumors: it acts as a tumor suppressor in breast cancer by silencing MEST to inhibit the Wnt/β-catenin pathway [39], while promotes genomic instability and aberrant cell cycle progression through BRCA1 regulation in ovarian cancer [51]. Additionally, its pivotal role in hematogenous metastasis of colorectal cancer has been well documented [52]. This functional heterogeneity might be attributed to the tissue-specific epigenetic regulatory mechanisms proposed by Olechnowicz et al.,whose team revealed that KRAB-ZFPs family members differentially modulate cancer stemness through distinct epigenetic modifications [53]. Notably, although the metabolic role of ZFP57 has remained elusive, our work provides the first evidence of its tumor-promoting mechanism in thyroid cancer CAFs through activating PKM2, a key glycolytic enzyme. We confirmed that ZFP57 directly binds to the PKM2 promoter region and enhances its transcriptional activity. These findings breakthroughly establish the central role of the ZFP57-PKM2 regulatory axis in CAFs-mediated metabolic reprogramming.

To bridge the gap between CAF-derived lactate and the nuclear reprogramming of thyroid cancer cells, we sought to identify the relevant signaling cascades that mediate this stromal-epithelial crosstalk.Traditionally regarded as a terminal product of glycolysis, lactate has been redefined as a multifunctional metabolic regulator within the tumor microenvironment [54, 55]. To mechanistically link CAF-derived lactate to the observed dedifferentiation phenotype, we investigated the TGF-β/Smad2/3 signaling pathway—a well-established cascade known to be modulated by lactate in other contexts. Our results confirm that in PTC, lactate indeed activates this pathway through MCT1/4-mediated transport, which correlates with the loss of thyroid differentiation markers. This finding extends the ‘lactate-TGF-β positive feedback loop’ theory to thyroid cancer [56], and suggests a potential signaling mechanism through which the metabolic microenvironment may influence epigenetic or transcriptional reprogramming. While previous studies have established the pro-tumorigenic roles of CAF-derived lactate in prostate cancer (invasion/metastasis) [57], colorectal cancer (metastatic potential) [58], and breast cancer (proliferation regulation) [59], our work highlights its potential role in modulating cell differentiation state, an aspect less explored previously. Furthermore, the “stromal metabolism-lactate shuttle-epithelial plasticity"axis proposed here may provide a mechanistic clue to the persistently activated TGF-β pathway observed in radioiodine-resistant thyroid cancer [60]. Notably, the dual functionality of lactate is fully demonstrated in this process: serving both as an alternative TCA cycle substrate to sustain energy metabolism [55], and functioning as a signaling molecule through epigenetic reprogramming mechanisms [61]. Additionally, the spatially heterogeneous expression patterns of MCT1/MCT4 observed in thyroid cancer microenvironment complement the classical “lactate shuttle” theorywith tissue-specific transport mechanism details [62].

As a low-toxicity natural polyphenolic compound [36, 63], resveratrol has demonstrated significant antitumor activity in both in vitro and in vivo models of ATC [42, 43].Its mechanisms of action encompass promoting tumor cell differentiation [64], inducing redifferentiation and apoptosis [65], and modulating thyroid cancer-associated signaling pathways. Our study extends these findings by first revealing that resveratrol reverses the glycolytic phenotype of CAFs through inhibition of the ZFP57-PKM2 axis, thereby restoring tumor cell differentiation capacity and enhancing radioactive iodine uptake. This discovery aligns closely with the ATA guideline’s emphasis on “differentiation status remodeling as the cornerstone of advanced thyroid cancer therapy” [66]. Compared with guideline-recommended multi-kinase inhibitors (e.g., sorafenib) that partially restore iodine uptake but exhibit significant toxicity [67, 68], resveratrol not only demonstrates superior safety profiles [69] but also exhibits a unique dual mechanism - simultaneously targeting CAF metabolic reprogramming and tumor cell redifferentiation. This provides novel insights for developing microenvironment-remodeling strategies. In accordance with ATA recommendations for molecular signature-guided interventions in radioiodine-refractory thyroid cancer [70], and considering recent breakthroughs in CAF-targeted imaging technologies [70, 71], our findings suggest the ZFP57/PKM2 pathway could serve as a biomarker for identifying PTC with dedifferentiation tendencies. Although resveratrol’s bioavailability limitations remain translational challenges [72], established nanoparticle delivery systems offer viable solutions for enhancing thyroid tumor specificity [73, 74]. Notably, preclinical studies reveal synergistic potential of resveratrol combination therapies: co-administration with rapamycin synergistically inhibits PI3K/AKT/mTOR signaling, significantly reducing PTC proliferation and key kinase phosphorylation levels [75]; combination with valproic acid downregulates ATC stem cell markers while enhancing differentiation marker expression [76]; concomitant use with retinoic acid improves differentiation indices (including thyroglobulin) by balancing CRABP2/RAR-β and FABP5/PPAR-β/δ signaling [42]. This “dual microenvironment-tumor targeting” strategy resonates with the combination therapy framework proposed by Gulley et al. [77], particularly suggesting potential synergy between the multi-targeted action of resveratrol (affecting metabolic reprogramming and differentiation) and kinase inhibitors like lenvatinib (targeting proliferation pathways) to achieve concurrent metabolic regulation and proliferation inhibition.While our data strongly support that resveratrol acts through inhibition of the ZFP57-PKM2 axis, we acknowledge that as a pleiotropic natural compound, it likely engages additional mechanisms to exert its full antitumor effects. The complete elucidation of its polypharmacology in reversing thyroid cancer dedifferentiation represents an important direction for future research.

### Limitations and future perspectives​​

This study has several limitations. First, the modest sample size of three patients may constrain the generalizability of our findings, particularly given the inherent challenges in expanding cohorts for ATC, a malignancy of extreme rarity. Second, while spatial transcriptomics provided valuable insights, its current resolution proved insufficient to delineate CAFs subpopulation heterogeneity. Additionally, although the lactate concentration employed (7.5 mM) falls within physiological parameters, direct measurement of CAF-secreted lactate levels through metabolomic profiling or microdialysis techniques would strengthen experimental validity. Furthermore, the observed defect in maximal respiratory capacity suggests a compromise in mitochondrial function that this study was unable to definitively address, warranting future ultrastructural analysis. Finally, the resveratrol dosage administered in animal models (100 mg/kg) raises translational concerns, as this regimen may not be clinically feasible. Optimization of dosing strategies and formulation development will be essential for potential human applications.

## Conclusion​​

This study elucidates the pivotal role of CAFs metabolic reprogramming in thyroid cancer dedifferentiation, mechanistically linking ZFP57-driven glycolysis with lactate-mediated Smad2/3 signaling activation. Our demonstration of resveratrol’s capacity to disrupt this axis provides a stromal-targeting strategy to reverse therapy-resistant dedifferentiation. Future investigations should validate the prognostic value of the ZFP57-PKM2 axis in expanded clinical cohorts and explore synergistic effects of resveratrol with differentiation-inducing agents, which could advance precision oncology frameworks for thyroid cancer management.

## Supplementary Information

Supplementary Material 1.

Supplementary Material 2.

Supplementary Material 3.

## Data Availability

The data that support the findings of this study are available from the corresponding author upon reasonable request.

## Abbreviations

AKT: Protein Kinase B
ATC: Anaplastic Thyroid Carcinoma
CAFs: Cancer-Associated Fibroblasts
CCK-8: Cell Counting Kit-8
ChIP: Chromatin Immunoprecipitation
CM: Conditioned Medium
DEGs: Differentially Expressed Genes
DDTC: Dedifferentiated Thyroid Carcinoma
ECAR: Extracellular Acidification Rate
EMT: Epithelial-Mesenchymal Transition
FFPE: Formalin-Fixed, Paraffin-Embedded
GEO: Gene Expression Omnibus
GO: Gene Ontology
H&E: Hematoxylin and Eosin
IL-6: Interleukin 6
JAK: Janus Kinase
KEGG: Kyoto Encyclopedia of Genes and Genomes
MAPK: Mitogen-Activated Protein Kinase
MCT1: Monocarboxylate Transporter 1
MCT4: Monocarboxylate Transporter 4
m6A: N6-Methyladenosine
mTOR: Mechanistic Target of Rapamycin
NIS: Sodium-Iodide Symporter
OCR: Oxygen Consumption Rate
PDTC: Poorly Differentiated Thyroid Carcinoma
PI3K: Phosphatidylinositol 3-Kinase
PKM2: Pyruvate Kinase M2
PPAR-β/δ: Peroxisome Proliferator-Activated Receptor Beta/Delta
PTC: Papillary Thyroid Carcinoma
RAR-β: Retinoic Acid Receptor Beta
Res: Resveratrol
Smad2/3: Mothers Against Decapentaplegic Homolog 2/3
STAT3: Signal Transducer and Activator of Transcription 3
ST: Spatial Transcriptomics
TGF-β: Transforming Growth Factor Beta
TME: Tumor Microenvironment
UMAP: Uniform Manifold Approximation and Projection
ZFP57: Zinc Finger Protein 57 (gene symbol)
