# Supplementary material for: Mechanism of cancer-associated fibroblast-driven thyroid cancer dedifferentiation via the ZFP57-PKM2 axis-mediated lactate secretion and therapeutic intervention with resveratrol​
Source: J Exp Clin Cancer Res. 2026 Feb 27;45:88. doi: 10.1186/s13046-026-03675-w (PMC13049851; doi:10.1186/s13046-026-03675-w)

**Figure S1**: **Spatial Transcriptomic Data Quality Assessment And Basic Analysis. (A-C)** Violin plots showing the distribution of gene counts, total UMI counts, and proportion of mitochondrial genes across all spots in different samples. The three sub - figures correspond to samples **(A)** TH - 1, **(B)** TH - 2, and **(C)** TH - 3. **(D-F)** Spatial visualization maps of genes and UMIs for different samples. The three sub - figures correspond to samples **(D)** TH - 1, **(E)** TH - 2, and **(F)** TH - 3. **(G-I)** Scatter plots showing the correlation between mitochondrial gene proportion, gene counts and UMI counts in different samples **(G)** TH - 1, **(H)** TH - 2, and **(I)** TH - 3. **(J)** PCA scree plot (x - axis: principal components; y - axis: the contribution of each principal component to explaining the original data). The three sub- figures correspond to samples

**Figure S2**: **Characteristics And Spatial Expression Patterns Of Cell Subpopulations. (A)** Visualization of sample cell subpopulations based on UMAP dimensionality reduction (different colors represent different cell subpopulations). **(B-D)** Expression of cell - type - specific marker genes in different samples. The dot size represents the proportion of cells expressing the gene, and the color gradient represents the log2(TPM+1) normalized expression level. The three sub - figures correspond to samples **(B)** TH - 1, **(C)** TH - 2, and **(D)** TH - 3. **(E)** Bar chart displaying the aggregated spatial distribution patterns of major cell types from three tissue sections. Colors correspond to different cell subsets..

**Figure S3**: **Functional Validation Of ZFP57-Mediated Metabolic Reprogramming In CAFs. (A)** Fluorescence in situ hybridization (FISH) identifying primary CAFs using α- SMA - specific probes. **(B)** shRNA - mediated knockdown efficiency of ZFP57, ZNF501, and SPIB in CAFs assessed by qPCR. **(C)** shRNA - mediated knockdown efficiency of ZFP57, ZNF501, and SPIB in CAFs assessed by Western blot. **(D)** Lactate production and glucose uptake in CAFs after ZFP57, ZNF501, and SPIB knockdown were measured using a lactate assay kit and a glucose assay kit, respectively. **(E)** Transfection efficiency of the Lv-ZFP57 lentiviral vector overexpressing ZFP57 in CAFs verified by qPCR. **(F)** Transfection efficiency of the Lv-ZFP57 lentiviral vector overexpressing ZFP57 in CAFs verified by Western blot. **(G)** Lactate production and glucose consumption in ZFP57-overexpressing CAFs were measured using a lactate assay kit and a glucose assay kit, respectively. **(H)** ECAR and OCR in ZFP57-knockdown CAFs were measured by the Seahorse XF Cell Energy Phenotype Test. **(I)** Representative TEM images of each group. The inset (black box, locally magnified area) shows morphologically altered mitochondria. Scale bars: main figure 1μm; inset 500 nm. For in vitro experiments or cell samples, n≥3 for each group. Data are mean ± SD. *p < 0.05, **p < 0.01, and ***p < 0.001. Statistical significance was determined by one-way ANOVA or two-sided Student’s t-test as appropriate.

**Figure S4**: **Spatial Transcriptomic Analysis Of ZFP57 And Metabolic Enzymes Co-Localization In Tumor Microenvironment CAFs. (A-H)** Co - localization analysis of metabolic enzymes with ZFP57 in CAFs across different differentiation regions of the tumor microenvironment based on spatial transcriptomic sequencing: ZFP57 with **(A)** HIF1A, **(B)** HK1, **(C)**HK2, **(D)** LDHA, **(E)** PFKM, **(F)** PFKP , **(G)** GLUT1**.** **(H)** Bar charts showing the number of co - localized spots of metabolic enzymes with ZFP57 in CAFs across different differentiation regions predicted by spatial transcriptomic sequencing.

**Figure S5: ZFP57 - Regulated Metabolic Reprogramming Of CAFs And Its Impact On Thyroid Cancer Cell Proliferation. (A)** qRT-PCR analysis of glycolysis-related enzyme (HIF-1α, PKM2, and PFKP) mRNA expression in CAFs after ZFP57 knockout and overexpression, respectively. **(B)** CCK - 8 assay for the proliferation of TPC1 cells co-cultured with conditioned medium from different CAFs groups. **(C)** qRT - PCR detection of thyroid - specific differentiation gene expression in thyroid cancer cells after ZFP57 overexpression in CAFs. **(D)** Radioactive iodine uptake in TPC1 and BCPAP cells treated with CAFs-CM under different conditions. **(E)** Transwell assay measuring the invasive capacity of TPC1 and BCPAP cells treated with CAFs-CM under different conditions.. For in vitro experiments or cell samples, n≥3 for each group. Data are mean ± SD. *p < 0.05, **p < 0.01, and ***p < 0.001. Statistical significance was determined by one-way ANOVA or two-sided Student’s t-test as appropriate.

**Figure S6: MCT4-Mediated Regulation Of Lactic Acid Transfer On Thyroid Cancer Cell Differentiation And Radiotherapy Sensitivity. (A)** Lactate production in CAFs after MCT4 knockdown detected by a lactate assay kit. **(B)** (A)Western blot analysis measuring the expression of thyroid differentiation proteins (NIS, TPO, and PAX8) in BCPAP cells treated with CAFs-CM under different conditions. **(C)** CCK-8 assay measuring the proliferation of BCPAP cells treated with CAFs-CM under different conditions. **(D)** Radioactive iodine uptake in BCPAP cells treated with CAFs-CM under different conditions. **(E)** Transwell assay measuring the invasive capacity of BCPAP cells treated with CAFs-CM under different conditions (scale bar: 100 µm). **(F)** CCK - 8 assay to determine the optimal lactic acid concentration. **(G)** Radioactive iodine uptake in BCPAP cells under different conditions is affected by treatment with CAFs-CM or lactate. **(H)** Transwell assay measuring the invasive capacity of BCPAP cells under different conditions is affected by treatment with CAFs-CM or lactate. **(I)** Western blot analysis measuring the expression of thyroid differentiation proteins (NIS, TPO, and PAX8) in BCPAP cells under different conditions is affected by treatment with CAFs-CM or lactate. **(J)** CCK-8 assay measuring the proliferation of BCPAP cells under different conditions is affected by treatment with CAFs-CM or lactate. **(K)**Western blot analysis of thyroid differentiation markers in xenografts. For in vitro experiments or cell samples, n≥3 for each group. Data are mean ± SD. *p < 0.05, **p < 0.01, and ***p < 0.001. Statistical significance was determined by one-way ANOVA or two-sided Student’s t-test as appropriate.

**Figure S7: Bioinformatic and experimental validation of lactate-associated pathways and phenotypes. (A)** GO pathway enrichment analysis of up-regulated differentially expressed genes from RNA-seq. **(B)** KEGG pathway enrichment analysis of up-regulated differentially expressed genes from RNA - seq. **(C)** GO pathway enrichment analysis of down-regulated differentially expressed genes from RNA-seq. **(D)** KEGG pathway enrichment analysis of down-regulated differentially expressed genes from RNA - seq. **(E)** GSEA of the epithelial compartment from spatial transcriptomics confirms significant enrichment of gene signatures for cell differentiation and TGF-β signaling in situ (FDR < 0.05). **(F)** Transwell assay measuring the invasive capacity of TPC1 and BCPAP cells under different conditions is affected by treatment with lactate. For in vitro experiments or cell samples, n≥3 for each group. Data are mean ± SD. *p < 0.05, **p < 0.01, and ***p < 0.001. Statistical significance was determined by one-way ANOVA or two-sided Student’s t-test as appropriate.

**Figure S8: Identification Of The Optimal Resveratrol Concentration. (A)** Lactate production in CAFs under different resveratrol concentrations detected by a lactate assay kit. **(B)** CCK - 8 assay to determine the optimal resveratrol concentration for CAFs. **(C)** Representative TEM images of each group. The inset (black box, locally magnified area) shows morphologically altered mitochondria. Scale bars: main figure 1μm; inset 500 nm. **(D)** Radioactive iodine uptake in TPC1 and BCPAP cells treated with conditioned medium from CAFs that were pretreated with resveratrol under different conditions. **(E)** Western blot analysis measuring the expression of NIS, TPO, and PAX8 in TPC1 and BCPAP cells treated with conditioned medium from CAFs that were pretreated with resveratrol under different conditions.. For in vitro experiments or cell samples, n≥3 for each group. Data are mean ± SD. *p < 0.05, **p < 0.01, and ***p < 0.001. Statistical significance was determined by one-way ANOVA or two-sided Student’s t-test as appropriate.

**
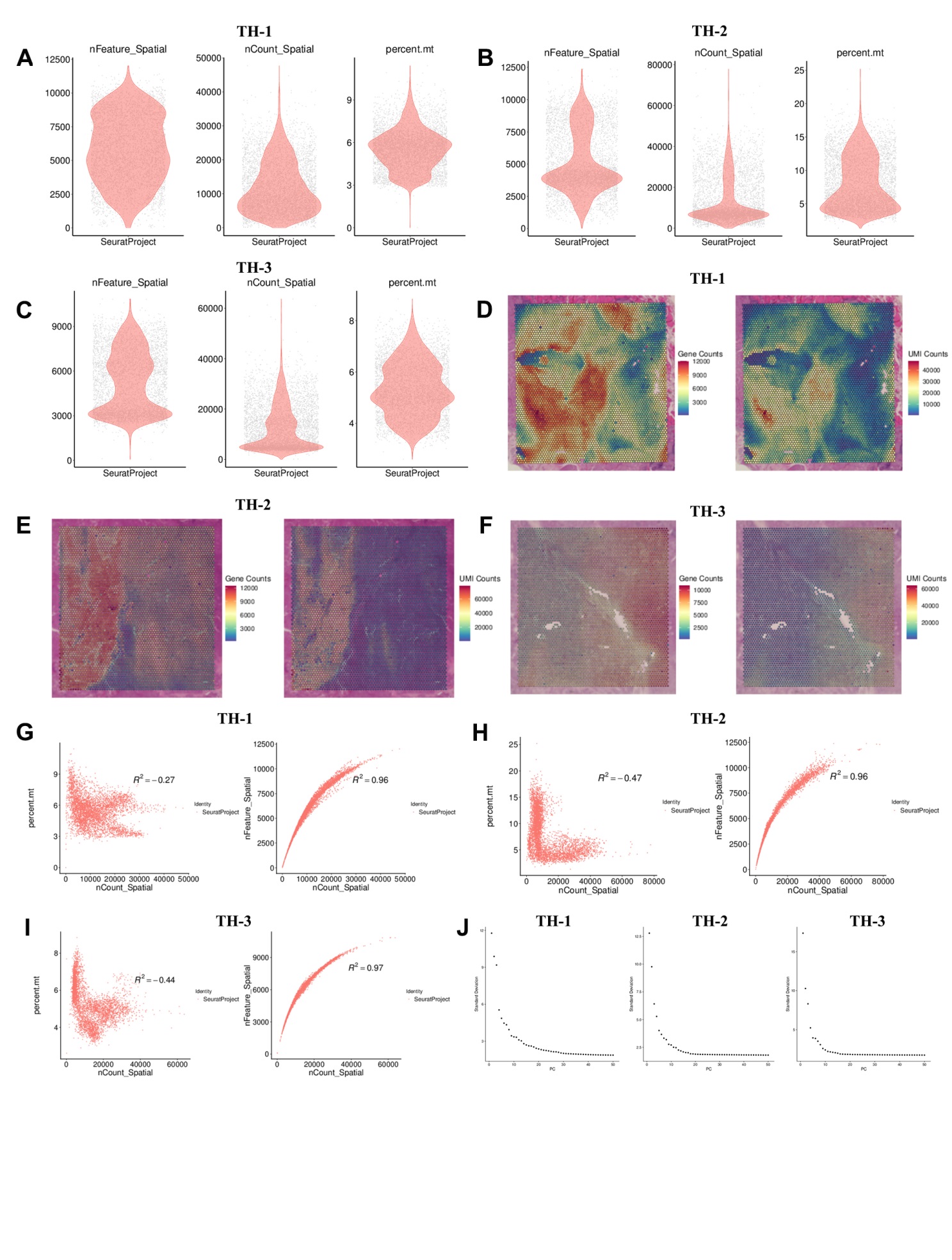

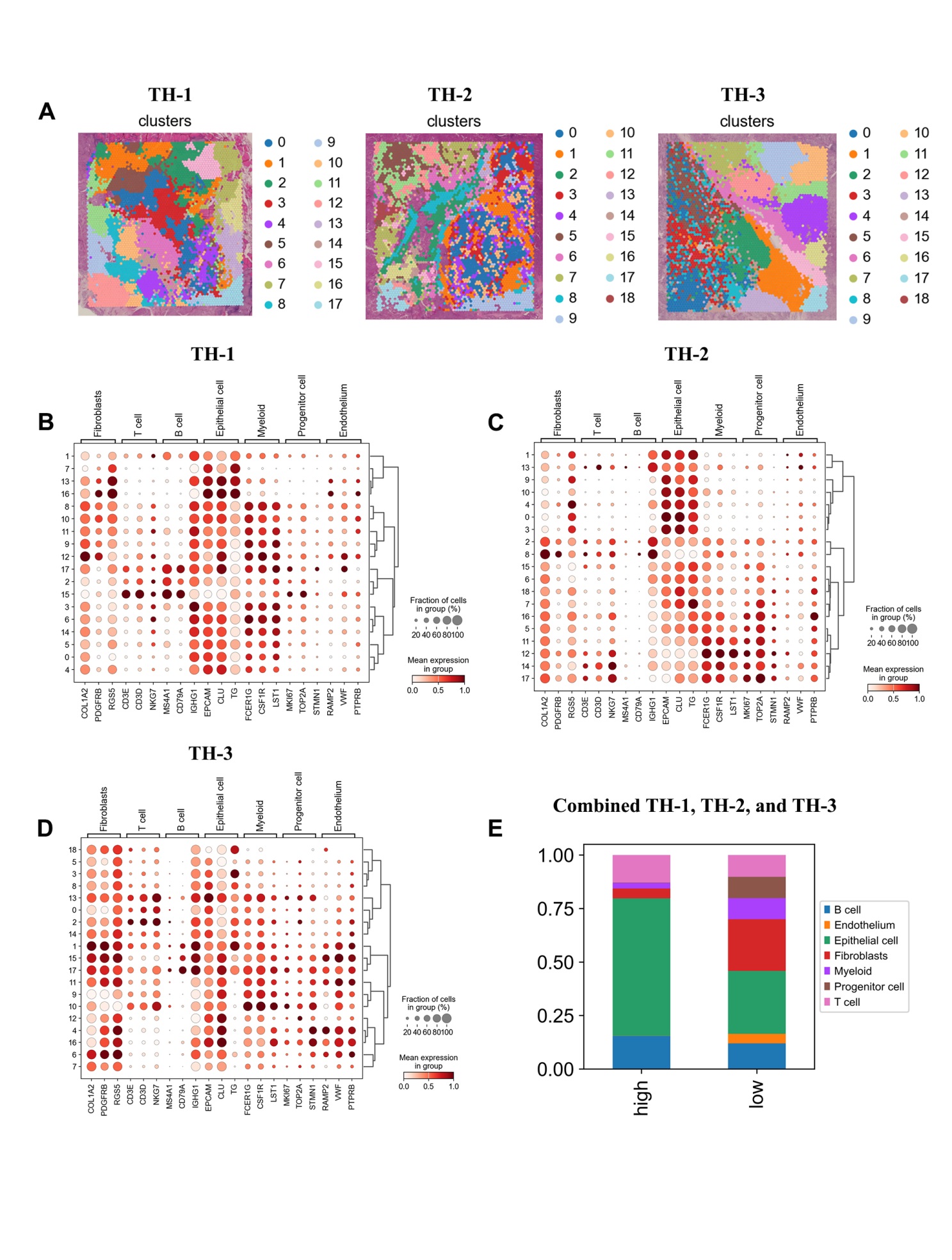

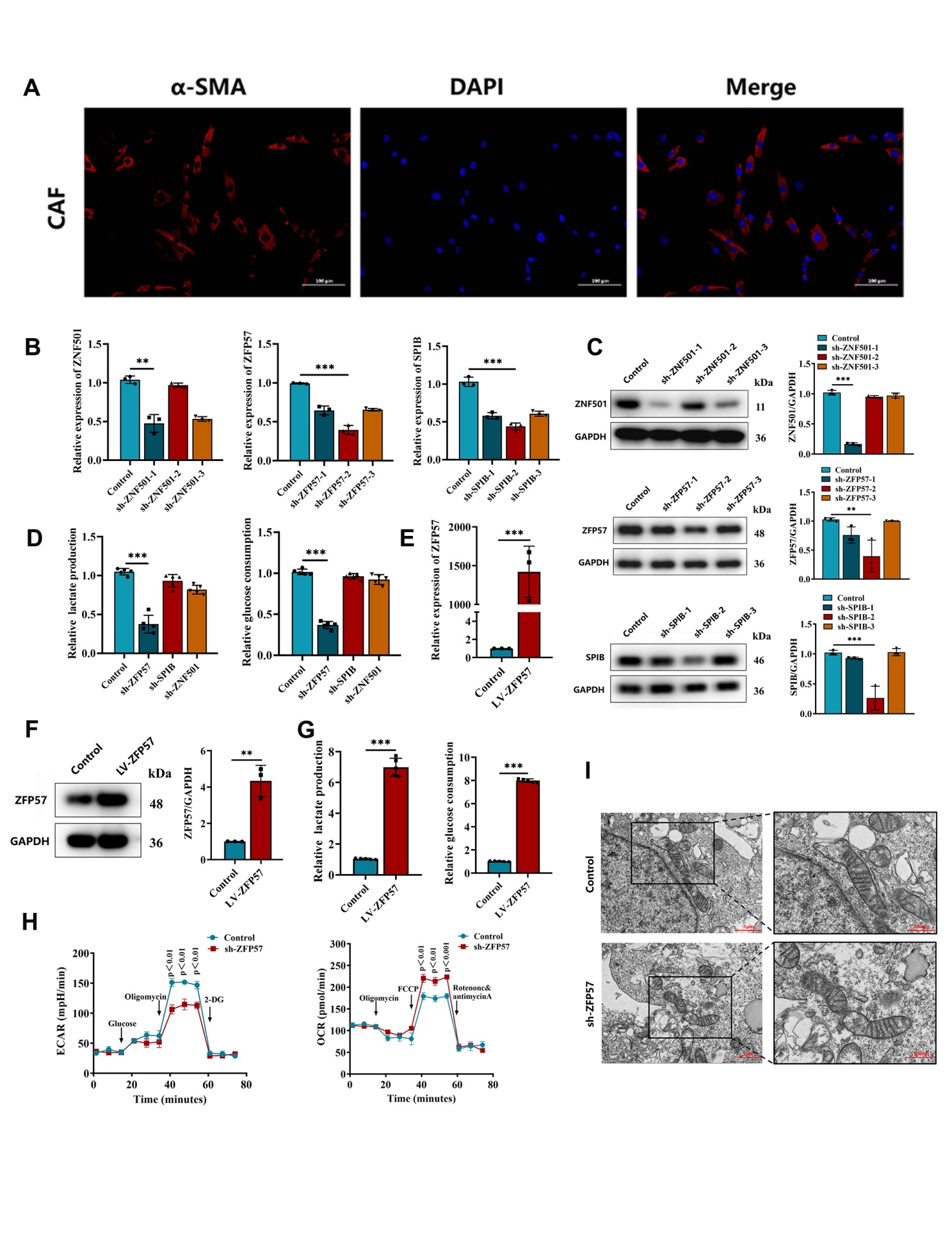

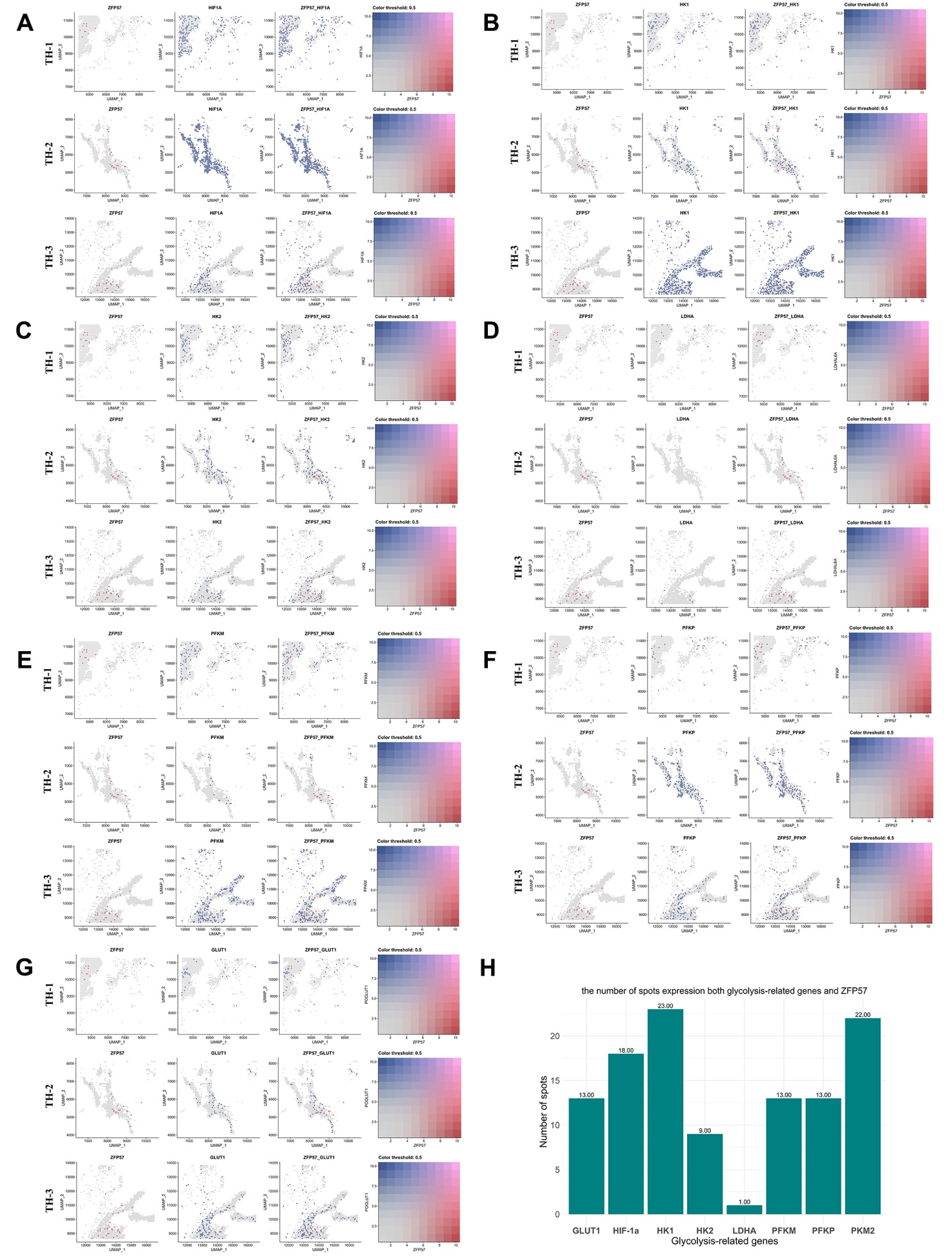
**


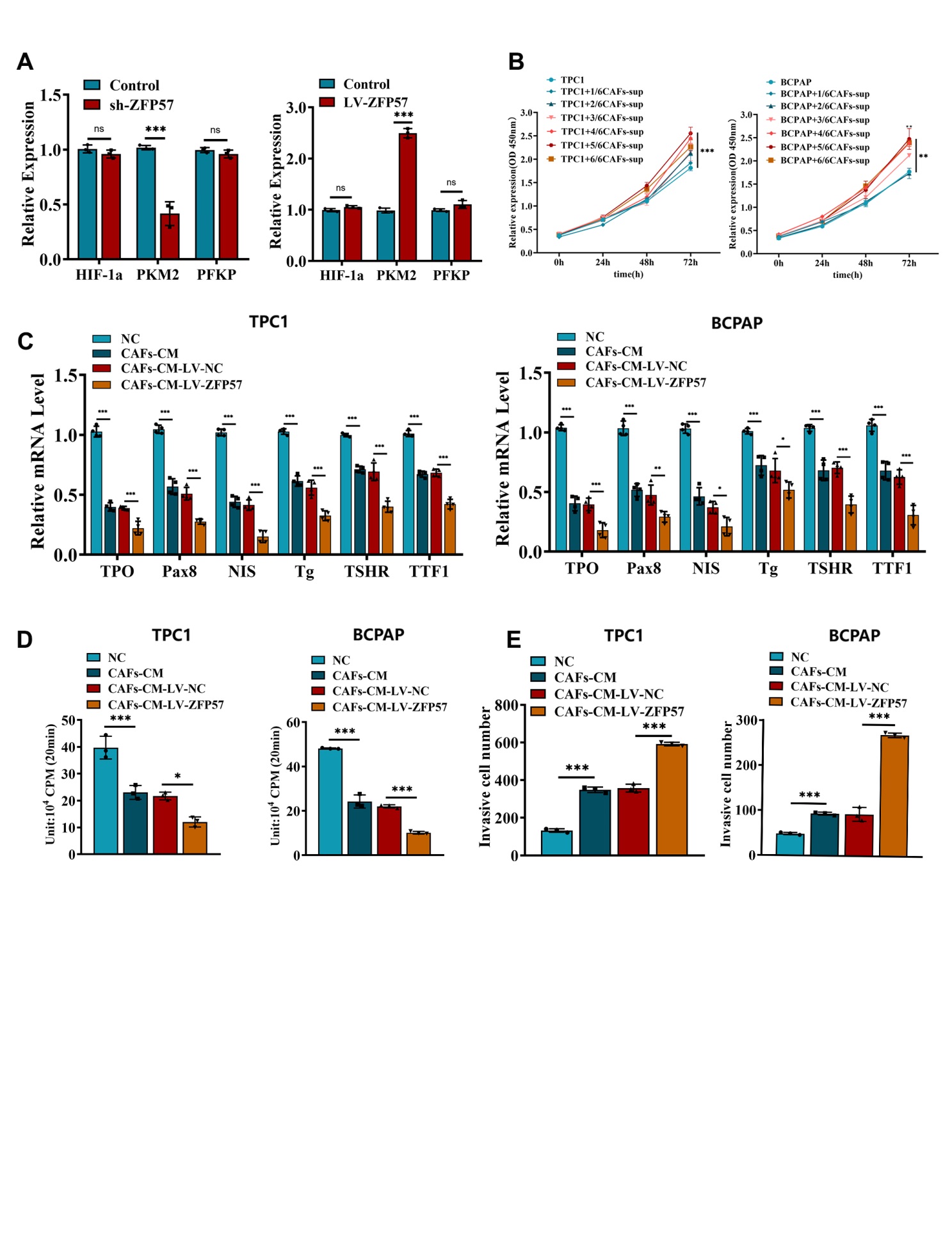

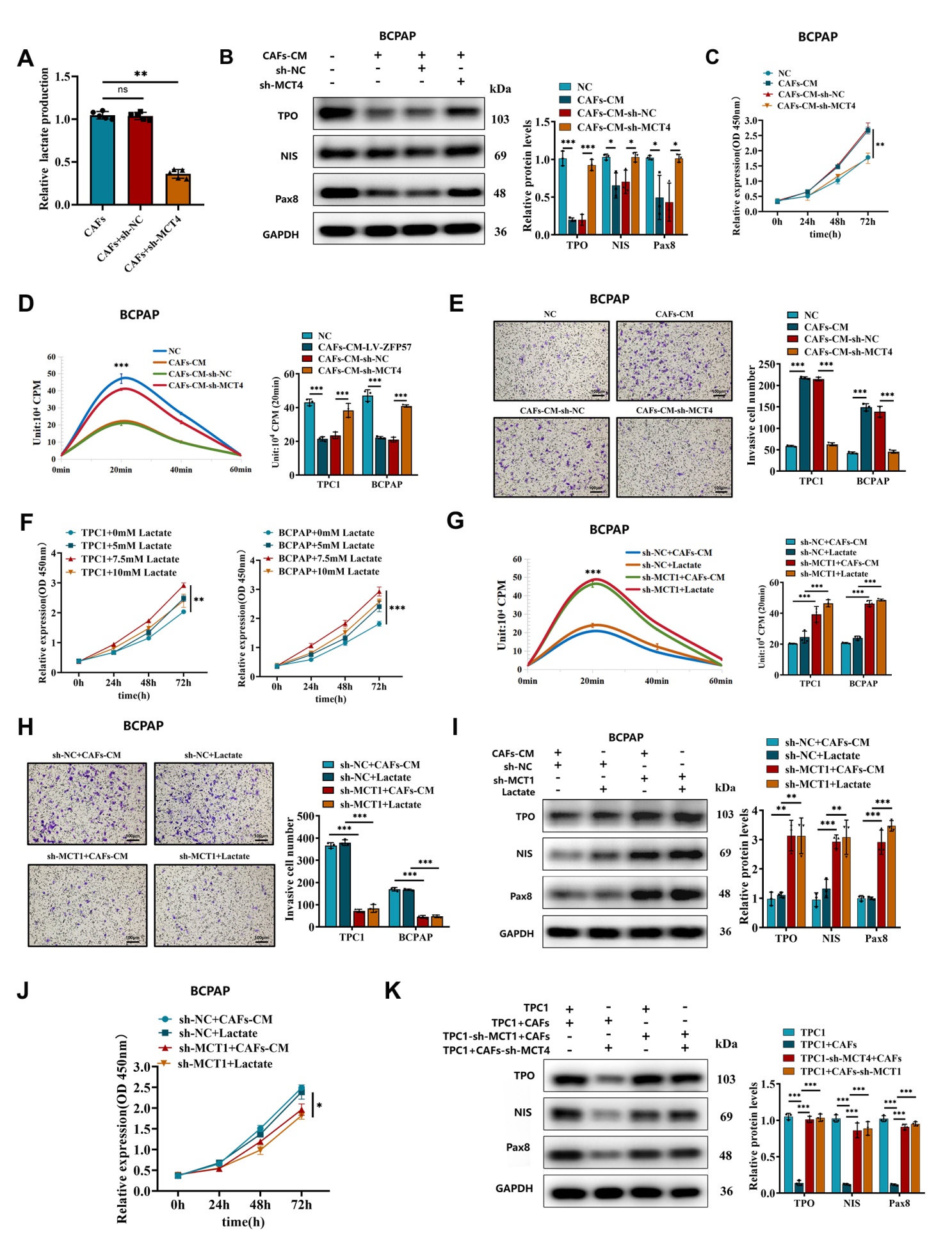

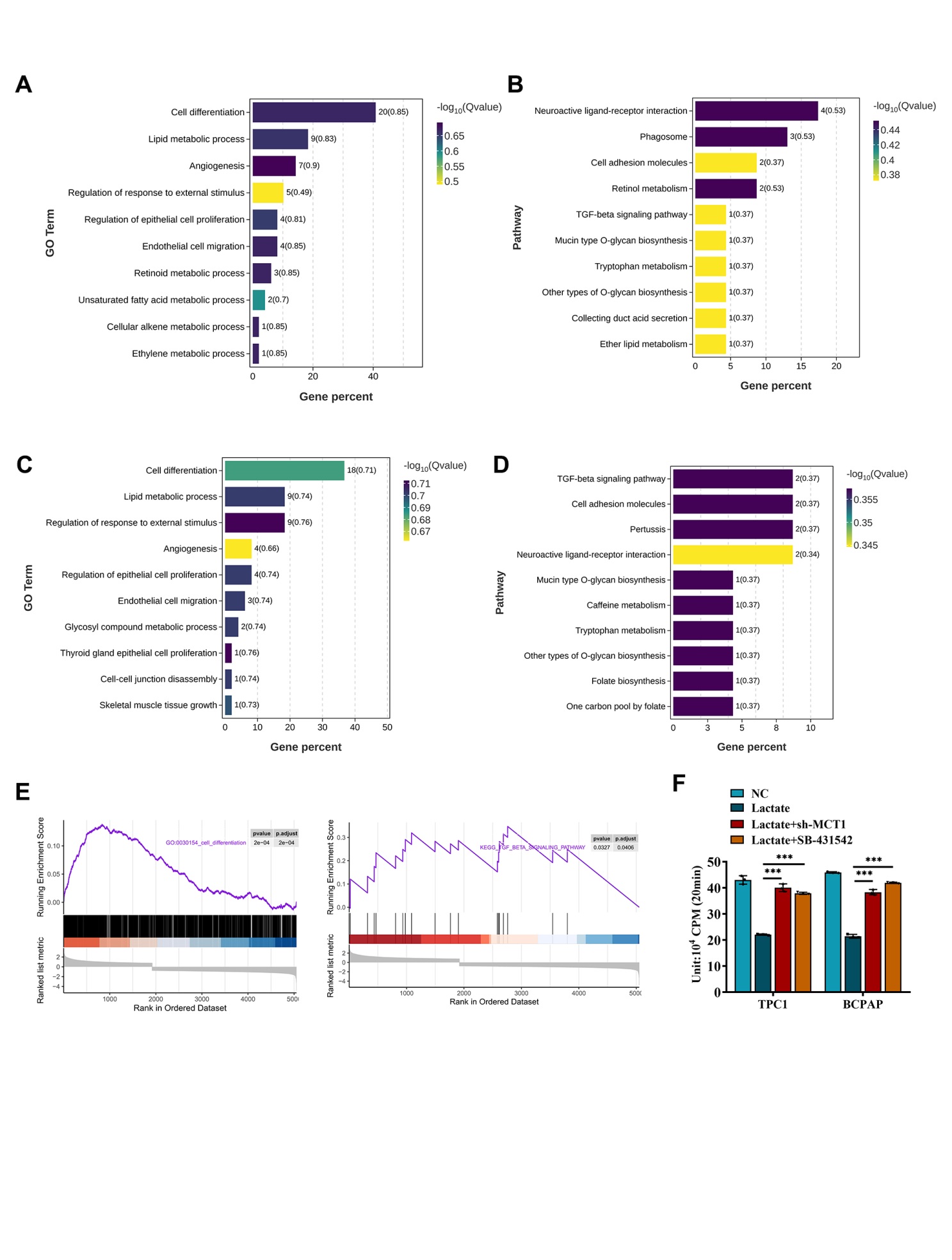

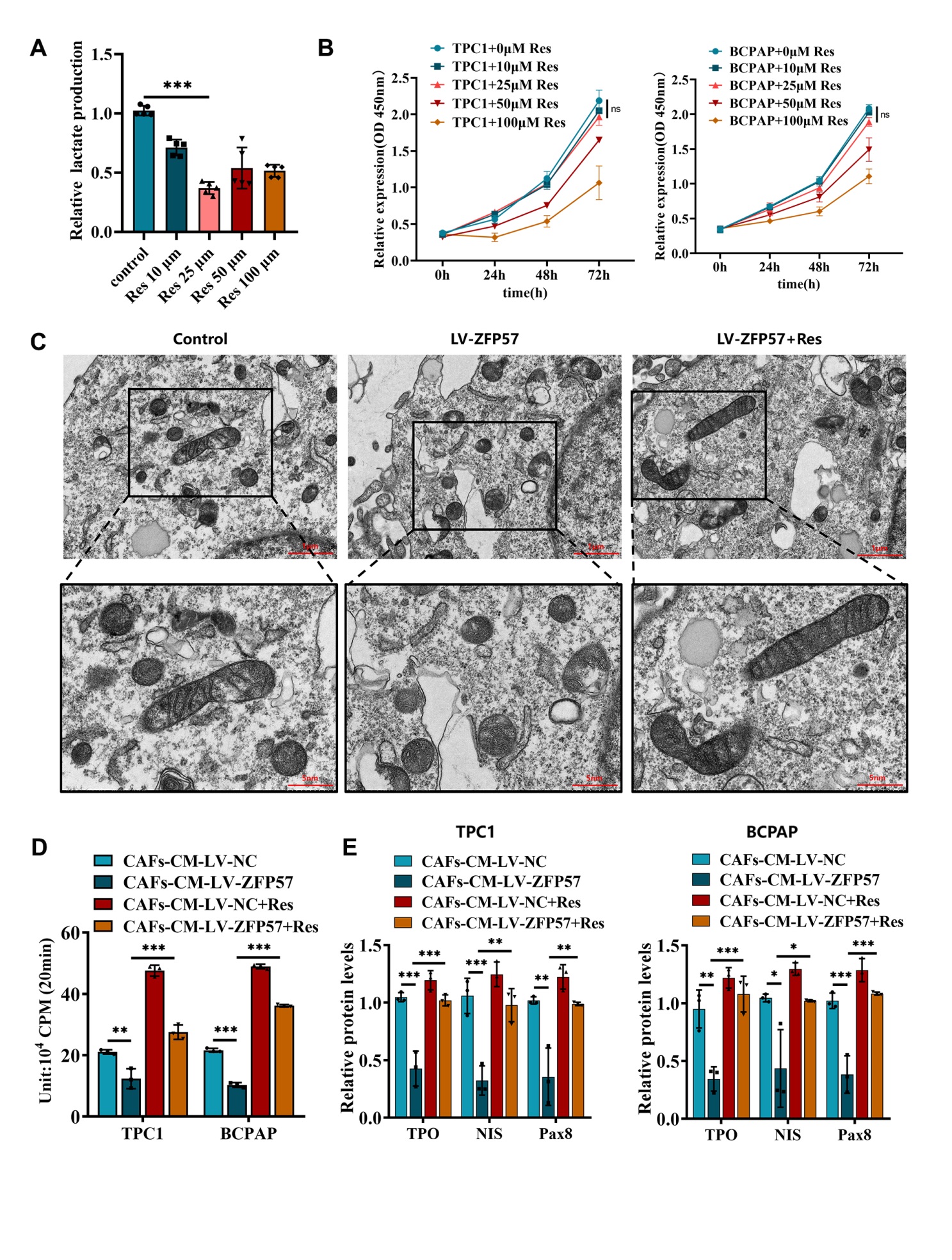

Supplement: Supplementary file 1 — Supplementary Material 1. [file 13046_2026_3675_MOESM1_ESM.docx]
