# Supplementary material for: Mechanism of cancer-associated fibroblast-driven thyroid cancer dedifferentiation via the ZFP57-PKM2 axis-mediated lactate secretion and therapeutic intervention with resveratrol​
Source: J Exp Clin Cancer Res. 2026 Feb 27;45:88. doi: 10.1186/s13046-026-03675-w (PMC13049851; doi:10.1186/s13046-026-03675-w)
Supplement: Supplementary file 3 — Supplementary Material 3. [file 13046_2026_3675_MOESM3_ESM.docx]

Methods

Supplementary Materials and Methods

**Cell Culture**

The human PTC-derived BCPAP and TPC1 cell lines were acquired from the National Collection of Authenticated Cell Cultures (Shanghai, China). The culture medium for BCPAP cells consisted of 44 mL RPMI 1640 basal medium, 0.5 mL non-essential amino acids, 0.5 mL sodium pyruvate, and 5 mL fetal bovine serum (FBS). TPC1 cells were cultured in 45 mL DMEM mixed with 5 mL FBS.

**Tissue Morphology Interpretation**

**Visium Spatial Tissue Optimization Protocol**

Spatial gene expression data for formalin-fixed paraffin-embedded (FFPE) tissues were obtained following the 10x Genomics protocols (CG000408, CG000409, and CG000407) for tissue preparation and library construction. Briefly, tissues were dewaxed, stained, and decrosslinked. Human whole transcriptome probe panel v1 was then hybridized to the dewaxed, stained, and decrosslinked tissues. Post-hybridization, probes were ligated, and ligation products were treated with RNase and permeabilized to release probes from the tissue. Ligated probe products were captured by spatially barcoded oligonucleotides and extended. Final libraries were generated from extended probes. Library concentration and quality were assessed using Qubit and Agilent TapeStation. Sequencing was performed on the Illumina NovaSeq 6000 platform with 28-bp reads for spatial barcodes and unique molecular identifiers (UMIs), and 50-bp reads for probe sequences.

**Demultiplexing and Gene Count Matrix Generation**

The Space Ranger software suite (v2.0.0) was used for gene expression alignment and quantification. Demultiplexing assigned spatial barcodes to reads. BCL files were converted to FASTQ format using Space Ranger mkfastq(), with low-quality reads filtered out. Sequencing data were aligned to the human reference genome (GRCh38) using the Space Ranger count module, with GENCODE v.32 as the gene annotation reference. Output matrices included UMI counts and gene expression counts per spatial barcode, corresponding to tissue regions with 55-μm diameter spots and 100-μm center-to-center spacing.

**Visium-Based Spatial Transcriptomics (ST) Data Analysis**

All spatial transcriptomics analyses were performed using Python (v3.10.14) and R (v4.3.3).

**Quality Control and Filtering**

Quality control and filtering were conducted using the Python package Scanpy (v1.9.7) for large-scale single-cell RNA-seq data. Filtering criteria included: 1) total counts per spot between 2,000 and 35,000; 2) genes detected in ≥10 spots; and 3) mitochondrial gene expression <20% per spot. High-quality spots were retained for downstream analyses.

**Batch Effect Correction and Cell Annotation**

Batch effects from three patient-derived datasets were corrected using the BBKNN algorithm (v1.6.0). Cell types were annotated using established marker genes, classifying spots into seven categories: fibroblasts (COL1A2, PDGFRB, RGS5), T cells (CD3E, CD3D, NKG7), B cells (MS4A1, CD79A, IGHG1), epithelial cells (EPCAM, CLU, TG), myeloid cells (FCER1G, CSF1R, LST1), progenitor cells (MKI67, TOP2A, STMN1), and endothelial cells (RAMP2, VWF, PTPRB).

**Plasmid Construction, Cell Transfection, and Lentiviral Infection**

Short hairpin RNAs (shRNAs) targeting ZFP57, SPIB, ZNF501, MCT1, and MCT4 were designed and cloned into lentiviral vectors. Non-targeting shRNA (Lv-NC) served as a negative control (Obio Technology). For ZFP57 overexpression, full-length coding sequences were cloned into lentiviral vectors (LV-ZFP57), with empty vectors as controls (Obio Technology, Shanghai, China). shRNA sequences are listed in Additional file 1（Table S1）

**Lentivirus Construction**

Short hairpin RNA (shRNA) sequences targeting human ZFP57, SPIB, ZNF501, MCT1, and MCT4 were designed and cloned into the pLKO.1-EGFP-puro lentiviral vector (HySigen Biosciences). A non-targeting shRNA (sh-NC) in the same vector served as a negative control. For overexpression, the full-length coding sequence (CDS) of human ZFP57 (NCBI Reference Sequence: NM_001109809.5) was synthesized and cloned into the pCDH-CMV-MCS-EF1-copGFP-T2A-Puro lentiviral vector (System Biosciences). An empty pCDH-CMV-MCS-EF1-copGFP-T2A-Puro vector was used as the control (Vector-NC). All oligonucleotide and shRNA sequences are listed in Additional file 1 (Table S1 and S2).

**Lentivirus transfection**

Based on pre-experimental results, BCPAP and TPC1 cells were transfected at multiplicity of infection (MOI) values of 30 and 40, respectively. Cells were seeded into 6-well plates at 1×10⁵ cells per well and cultured for 18–24 hours. After washing with PBS, medium containing P infection reagent and the corresponding volume of virus solution was added. The plates were gently mixed and incubated at 37°C under 5% CO₂. After 12 hours, fresh medium was replaced. GFP expression was observed under a fluorescence microscope 24 hours post-infection to assess infection efficiency. Puromycin was then used for selection, with drug-containing medium replaced every 2–3 days for approximately one week to obtain stably transfected cell lines.

**Total RNA Isolation and qRT-PCR**

Total RNA was extracted using RNAiso (Takara, Dalian, China) and reverse-transcribed into cDNA with the PrimeScript RT Reagent Kit (RR036A, Takara, Japan). qRT-PCR was performed using SYBR Premix Ex TaqII (Takara) on a LightCycler 480 system (Roche, USA). Amplification conditions: 95°C for 30 sec, followed by 50 cycles of 95°C for 5 sec and 60°C for 30 sec, with dissociation at 95°C (60 sec), 55°C (60 sec), and 95°C (30 sec). miRNA primers were included in the Mir-X miRNA First-Strand Synthesis Kit (638313, Takara). Relative expression was calculated using the 2^−ΔCT method, with GAPDH and U6 as internal controls. Primer sequences are listed in Additional file 1（Table S2）.

**Western Blotting**

Total protein was extracted using lysis buffer, and concentrations were measured with a BCA Protein Assay Kit (Beyotime, China). Proteins were separated by 10% or 12% SDS-PAGE and transferred to PVDF membranes (Bio-Rad, USA). Membranes were blocked with 5% non-fat milk for 2 hr, incubated with primary antibodies at 4°C overnight, and then with HRP-conjugated secondary antibodies (1:20,000) for 2 hr. Protein bands were visualized using an enhanced chemiluminescence system.Antibody brands are listed in Additional File 1 (Table S3).

**Cell Proliferation Assay**

Cell proliferation was assessed using the CCK-8 Kit (Dojindo, Japan). Cells (2×10^3/well) were seeded in 96-well plates and allowed to adhere overnight. The next day (designated as time 0 hr), the medium was replaced with treatment mediums (e.g., CAF-CM or control medium). After treatment, 10 μL CCK-8 reagent was added to each well at 0, 24, 48, and 72 hr, followed by 3 hr incubation at 37°C. Absorbance at 450 nm was measured to quantify viable cells.

**Transwell Invasion Assay**

Cells were resuspended in serum-free medium at a density of 1×10⁵ cells/mL. Then, 200 µL of the cell suspension (containing 2×10⁴ cells) was carefully added to the upper chamber of each Matrigel-coated Transwell insert. The lower chamber was filled with 600 µL of complete medium containing 10% FBS as a chemoattractant. After incubation for 24 hours at 37°C in a 5% CO₂ atmosphere, non-invasive cells on the upper surface of the membrane were gently removed with a cotton swab. Cells that had invaded through the Matrigel and membrane to the lower surface were fixed with 4% paraformaldehyde, stained with 0.1% crystal violet, imaged, and counted under a light microscope. Experiments were performed in triplicate.

**Immunofluorescence (IF)**

Cancer-associated fibroblasts (CAFs) were identified by α-smooth muscle actin (α-SMA) expression. Cells were fixed, permeabilized, and stained with anti-α-SMA antibody (1/500, ab5694, Abcam, UK) and HRP-conjugated secondary antibody (1/2000, ab205718, Abcam).

**Multiplex Immunofluorescence (mIF)**

Cells grown on coverslips were fixed, permeabilized with 0.5% Triton X-100, and blocked with 5% BSA. Primary antibodies (1:200 dilution) were applied overnight at 4°C, followed by species-specific secondary antibodies and DAPI nuclear staining. Images were acquired using fluorescence microscopy.

**TEM Sample Preparation and Imaging**

For transmission electron microscopy (TEM) analysis, cell or bacterial samples were collected by centrifugation and fixed in glutaraldehyde-based fixative at 4 °C for 2–4 h. After washing in phosphate buffer, the pellets were pre‑embedded in 1 % agarose and post‑fixed with 1 % osmium tetroxide. Dehydration was performed using a graded ethanol series followed by acetone, and samples were infiltrated and embedded with 812 epoxy resin. Polymerization was carried out at 60 °C for 48 h. Ultrathin sections (60–80 nm) were cut using an ultramicrotome, collected on copper grids, and double‑stained with 2 % uranyl acetate and 2.6 % lead citrate. Observations and image acquisition were performed using a Hitachi HT7800/HT7700 transmission electron microscope.
